# Prevalence and genetic basis of extended-spectrum β-lactamase-producing *Escherichia coli* carriage in broiler farms in the United Arab Emirates

**DOI:** 10.3389/fvets.2025.1714381

**Published:** 2025-12-08

**Authors:** Hazim O. Khalifa, Temesgen Mohammed, Mohammed Elbediwi, Afra Abdalla, Mohamed-Yousif Ibrahim Mohamed, Hazem Ramadan, Glindya Bhagya Lakshmi, Akela Ghazawi, Ihab Habib

**Affiliations:** 1 Department of Veterinary Medicine, College of Agriculture and Veterinary Medicine, United Arab Emirates University, Al Ain, United Arab Emirates; 2 Institute for Medical Microbiology and Virology, Carl von Ossietzky University Oldenburg, Oldenburg, Germany; 3 Animal Health Research Institute, Agriculture Research Centre, Cairo, Egypt; 4 ASPIRE Research Institute for Food Security in the Drylands (ARIFSID), United Arab Emirates University, Al Ain, United Arab Emirates; 5 Hygiene and Zoonoses Department, Faculty of Veterinary Medicine, Mansoura University, Mansoura, Egypt; 6 Department of Medical Microbiology and Immunology, College of Medicine and Health Sciences, United Arab Emirates University, Al Ain, United Arab Emirates

**Keywords:** ESBL, antimicrobial resistance, Gram-negative bacteria, chicken cecum, poultry farms, E. coli, animal-to-human transmission

## Abstract

**Background:**

Poultry production plays a vital role in ensuring food security and nutritional sustainability in the United Arab Emirates (UAE). However, the emergence and spread of antimicrobial-resistant bacteria in poultry farms present a growing public health concern in the region. To address this gap, this study investigated the prevalence and genetic basis of extended-spectrum β-lactamase (ESBL)-producing Gram-negative bacteria in cecal contents of chickens collected from two major poultry farms in Al Ain, UAE.

**Methods:**

A total of 77 samples were collected over an eleven-month period, yielding 146 non-duplicate Gram-negative isolates, of which *Escherichia coli* was the most prevalent species (82.9%). All the isolates were tested phenotypically and genotypically by PCR and whole genome sequencing for different resistance mechanisms.

**Results:**

Phenotypic characterization revealed high rates of ESBL production (87.7%), with 95% of *E. coli* isolates exhibiting this trait. Antimicrobial susceptibility testing indicated high resistance among β-lactam antibiotics, with ampicillin (100%), ceftriaxone (91.1%), cefoperazone (89%), and cefoxitin (48.6%), while resistance to amoxicillin-clavulanic acid (10.3%), meropenem (0%), and imipenem (0%) was notably lower. Overall resistance rates were also high for quinolones (89%), tetracycline (72.6%), and chloramphenicol (41.1%). The genotypic analysis identified *bla*_TEM_ (78.1%) and *bla*_CTX−M_ (76.7%) as the most common resistance genes, with *bla*_CTX−M−1_ group being predominant. Whole-genome sequencing of selected isolates confirmed the presence of *bla*_CTX−M−55_, *bla*_CTX−M−15_, and *bla*_CTX−M−8_ as major genes contributing for ESBL production along with various other resistance and virulent determinants. Notably, 10.3% of isolates harbored the mcr-1.1 gene, indicating colistin resistance. WGS-based sequence typing revealed 19 distinct sequence types (STs), with ST694 being the most prevalent, followed by ST10 and ST155. Several isolates, such as ST162 and ST10 were recovered from different farms, suggesting possible dissemination across poultry production sites. Phylogenetic analysis revealed that the multidrug-resistant *E. coli* lineages identified in this study were highly related to isolates recovered from humans and chicken carcasses in the UAE, indicating possible transmission events within and between poultry farms.

**Conclusion:**

Our findings underscore the potential zoonotic risk posed by these strains and highlight the urgent need for strengthened antimicrobial stewardship, enhanced biosecurity practices, and continuous surveillance to limit the spread of resistant bacteria in poultry production and protect public health.

## 1 Introduction

Antimicrobials, hailed as one of the most revolutionary medical breakthroughs of the 20th century, have saved countless lives and are widely used not only in human healthcare but also in animal husbandry and production. However, decades of misuse and overuse in both sectors have led to the selection of resistant bacteria, exacerbating the antimicrobial resistance (AMR) crisis. Without immediate action, AMR-related deaths are projected to rise to 10 million annually by 2050, surpassing mortality rates from cancer (1). According to a major study published in 2022, bacterial AMR was associated with approximately 4.95 million deaths in 2019, with 1.27 million of these directly attributed to resistant infections (2). Regions such as western Sub-Saharan Africa experienced the highest mortality rates, with 27.3 deaths per 100,000 people (2). The six leading pathogens responsible for resistance-related deaths—*Escherichia coli, Staphylococcus aureus, Klebsiella pneumoniae, Streptococcus pneumoniae, Acinetobacter baumannii*, and *Pseudomonas aeruginosa*—together accounted for over 3.5 million AMR-associated deaths (2).

Poultry farming plays a vital role in ensuring food security and nutrition, particularly in developing countries. Therefore, the issue of AMR becomes even more critical when it intersects with the food chain, exacerbating the ongoing global food crisis that impacts millions worldwide. Moreover, poultry farms are frequently associated with zoonotic foodborne pathogens, such as extended-spectrum β-lactamase (ESBL)-producing bacteria, which are among the most commonly reported threats to public health (3). ESBLs are enzymes that render bacteria resistant to most beta-lactam antibiotics, including penicillins, cephalosporins, and the monobactams (4, 5). Identifying ESBL-producing pathogens in the food supply chain is essential due to the critical importance of β-lactam antibiotics in both human and veterinary medicine (6, 7). These antibiotics are widely utilized because of their highly effective therapeutic properties. The World Health Organization (WHO) has classified several β-lactam antibiotic classes, such as carbapenems and third-, fourth-, and fifth-generation cephalosporins, as “critically important” for human health (8). Moreover, in some developing countries, antibiotics originally intended for human medicine, including those prohibited in veterinary practice, have been used in animals to enhance treatment outcomes (4, 5, 9). This widespread application of β-lactams across human and veterinary sectors has led to significant selection pressure, facilitating the emergence and spread of resistance in humans, animals, and the food chain (9–11).

Checking ESBL-producing bacteria in the chicken cecum is important due to its implications for public health, food safety, and AMR monitoring. The chicken cecum serves as a reservoir for a diverse microbial population, including potential zoonotic pathogens such as *Escherichia coli* and *Klebsiella pneumoniae* that can harbor ESBL genes (12). The detection of ESBL-producing bacteria in the cecum is significant because improper evisceration during processing can lead to leakage of intestinal contents, contaminating the surface of broiler carcasses and thereby increasing the risk of human exposure (13, 14). Furthermore, the cecum's environment, rich in nutrients and conducive to bacterial survival, facilitates the proliferation and exchange of resistance genes via horizontal gene transfer (15). Surveillance of ESBL-producing bacteria in chicken cecum is, therefore, essential to identify potential health risks, guide antibiotic use policies in poultry farming, and mitigate the spread of AMR through the food chain. In the UAE, there is limited information regarding the prevalence and genetic characteristics of ESBL-producing Gram-negative bacteria in poultry farming. To address this gap, this study was designed to assess the prevalence, phenotypic traits, and genetic resistance profiles of ESBL-producing Gram-negative bacteria in chicken cecal samples. Additionally, for the first time, it explores the genetic relatedness between ESBL-producing *Escherichia coli* isolated from chicken cecum and those recovered from other sources, including humans, animals, food, and the environment in the UAE. Specifically, this study aims to determine the prevalence of ESBL-producing *E. coli* in poultry cecal samples, identify the major ESBL and antimicrobial resistance genes carried by these isolates, and evaluate whether these strains share close genetic relationships with local clinical, food, and environmental isolates, thereby suggesting potential cross-sector or zoonotic transmission.

## 2 Materials and methods

### 2.1 Sample details

In this study, a total of 77 samples yielding 146 non-duplicated isolates were collected over an eleven-month period (June 2023 to April 2024) from various farmhouses and slaughterhouses operated by two major poultry companies in Al Ain City, UAE. The extended sampling period increases the representativeness and robustness of the findings, as it encompasses different production batches and seasonal variations in poultry operations. The samples comprised 59 cecal droppings and 18 cecal content samples. To ensure diversity in sampling, specimens from the first company were obtained from 18 poultry houses across six different farms, while samples from the second company were collected from nine houses located in three distinct farms. The sampling procedure was conducted as previously described in detail by Habib et al. (16). For the cecal drop samples, approximately 250 g of dark feces (excreted from the cecum) were collected in a sterile tube and transported to the laboratory under aseptic and cooled conditions. Upon arrival at the lab, the samples were thoroughly mixed, and 100 g of feces were combined with 100 ml of sterile peptone water and processed in a stomacher for 1 min. Next, 50 ml of this mixture was transferred to a sterile stomacher bag and mixed with 200 ml of sterile peptone water, followed by an additional minute of stomaching. The resulting mixture was then serially diluted ten times in sterile buffered peptone water. From each dilution, streaking was performed onto MacConkey agar containing 4 μg/ml of cefotaxime. For cecal content isolation, the cecal content was collected from 15 freshly slaughtered chickens in sampled slaughterhouses. The cecal contents were collected directly into sterile tubes and transported to the lab under aseptic, cooled conditions. Upon arrival, the samples were mixed, and 5 g of the contents were thoroughly mixed with 45 ml of buffered peptone water. The sample underwent serial dilution, and 100 μl of each dilution was plated onto MacConkey agar containing 4 μg/ml of cefotaxime.

### 2.2 Bacterial isolation and characterization

After plating on MacConkey agar supplemented with 4 μg/ml of cefotaxime, all plates were incubated at 37 °C for 24 h. Specific colonies that varied in size and/or color were selected from each sample, streaked onto nutrient agar, and incubated at 37 °C for another 24 h. These isolates were then preserved in 25% glycerol with tryptic soy broth at −80 °C. The identification and confirmation of all isolates were performed using Matrix-assisted laser desorption/ionization time-of-flight mass spectrometry (MALDI-TOF-MS), as described in previous studies (4, 5).

### 2.3 Antimicrobial susceptibility testing

As outlined by the Clinical and Laboratory Standards Institute (17), the antimicrobial susceptibility of the isolates was assessed using the Kirby–Bauer disc diffusion method, with *E. coli* ATCC 25922 serving as the quality control strain. In this study, a range of antibiotic discs (HiMedia, Maharashtra, India) representing different antibiotic classes were tested, including β-lactams [mpicillin (AMP), 10 μg; amoxicillin–clavulanic acid (AMC), 20–10 μg; cefoperazone (CFP), 75 μg; ceftriaxone (CRO), 30 μg; cefoxitin (FOX), meropenem (MEM), 10 μg and 30 μg; imipenem (IPM), 10 μg], quinolones [ciprofloxacin (CIP), 5 μg and nalidixic acid (NAL), 30 μg], aminoglycosides [gentamicin (GEN), 10 μg and amikacin (AMK), 30 μg], tetracycline (TET), 30 μg, and chloramphenicol (CHL), 30 μg. Antibiotic susceptibility results were interpreted according to the CLSI clinical breakpoints outlined in document M100-S30 (17).

### 2.4 Phenotypic detection of extended-spectrum β-lactamase production

Phenotypic confirmation of extended-spectrum β-lactamase (ESBL) production was performed using the CLSI confirmatory double disc synergy test (DDST). This test involved placing ceftriaxone (CTA; 30 mg) and ceftazidime (CAZ; 30 mg) discs both individually and in combination with clavulanic acid (CA; 10 mg) (HiMedia, Maharashtra, India). A positive result was indicated if the zone of inhibition around either the CTA or CAZ disc, when combined with clavulanic acid, was 5 mm or more larger than the zone around the disc containing CTA or CAZ alone.

### 2.5 DNA preparation and PCR experiments

The DNA was extracted using a boiling lysate method as previously described (4, 5). In brief, a 1-μl loopful of an overnight bacterial culture grown on Mueller–Hinton agar (MHA) was suspended in 100 μl of sterile distilled water and then boiled for 8 min to release the DNA. The resulting bacterial suspension was then shaken thoroughly, centrifuged, and the supernatant (2-μl) was used as a template for PCR analysis. Genetic characterization of the isolates was performed through PCR, as outlined in previous studies (4, 18–21). The isolates were screened for a variety of β-lactam resistance genes using single or multiplex PCR, as described in the Supplementary Table S1 (4, 18–21). For the detection of *bla*_CTX−M_, *bla*_SHV_, *bla*_OXA_, and *bla*_TEM_, multiplex PCR was employed, and all *bla*_CTX−M_ positive isolates were tested to identify groups one, two, and nine using multiplex PCR (4, 18–21).

### 2.6 Whole genome sequence (WGS) analysis

Whole-genome sequencing (WGS) was performed on 31 representative *E. coli* isolates, including 29 ESBL-producing and 2 non-ESBL-producing strains, selected based on their antimicrobial resistance patterns, sample origin, and relevance to the study objectives. This approach aimed to validate the phenotypic findings and gain a deeper understanding of the molecular mechanisms of resistance. Genomic DNA was isolated using the Wizard^®^ Genomic DNA Purification Kit (Promega, Madison, WI, USA), following the protocol provided by the manufacturer. DNA yield and purity were assessed using two spectrophotometric instruments—the ScanDrop2 nano-volume spectrophotometer (Analytikjena, Life Science, Germany) and the NanoDrop 1000 UV-Vis spectrophotometer (Thermo Scientific, Waltham, MA, USA)—to cross-verify the accuracy of DNA quantification and quality measurements prior to WGS. WGS libraries were prepared using the Illumina Nextera XT kit (Illumina, USA), generating paired-end reads with a length of 150 base pairs. Sequencing was carried out using Illumina's NovaSeq short-read platform by Novogene, a professional sequencing service provider based in the United Kingdom. The resulting raw reads underwent quality assessment and *de novo* assembly using SPAdes version 4.0.1. The assembly process included the ‘careful' mode to reduce the occurrence of mismatches, with k-mer sizes automatically selected by the software (22). In addition, virulence genes, antimicrobial resistance genes, and plasmid types were identified using the ABRicate tool (https://github.com/tseemann/abricate, accessed on March 1st, 2025), with a minimum identity threshold of 90% and minimum coverage of 60%. The heatmaps and hierarchical clustering were done using pheatmap 1.0.12 package (10.32614/CRAN.package.pheatmap) in R software. To place our isolates within the context of local *E. coli* lineages, we performed core-genome multilocus sequence typing (cgMLST) in combination with single-nucleotide polymorphism (SNP) analysis, following the approach described by Zhou et al. (23). The isolates from this study were uploaded to EnteroBase (https://enterobase.warwick.ac.uk/species/index/ecoli) and compared against 112 publicly available *E. coli* genomes from the UAE, representing a range of sources (Supplementary Table S2). SNP analysis was conducted using *E. coli* K-12 MG1655 as the reference genome, and the isolates were categorized into HC200 clusters, defined as having ≤200 allele differences in the core genome. Metadata for the selected *E. coli* isolates retrieved from EnteroBase are summarized in Supplementary Table S2.

Plasmid reconstruction for *E. coli* isolates carrying the *bla*_CTX−M−8_ gene was performed using MOB-Recon (24). The resulting plasmid sequences were aligned against the NCBI database to identify the most similar plasmid matches. Genome annotation of the reconstructed plasmids was carried out using Prokka (25). Comparative genomic analysis between the reconstructed plasmids and those retrieved from NCBI was visualized using the BLAST Ring Image Generator (BRIG) tool (https://sourceforge.net/projects/brig/).

## 3 Results

### 3.1 Isolates characterization and ESBL production

In this study, a total of 146 non-duplicate Gram-negative isolates were recovered from 77 tested samples. Species identification revealed that the majority of isolates (82.4%, 121/146) belonged to the *Escherichia* genus, with *E. coli* being the most prevalent species (82.2%, 120/146), along with a single *E. vulneris* isolate. In addition, 13 isolates (8.9%) were identified as *Salmonella* spp., nine (6.2%) as *Klebsiella pneumoniae*, two (1.4%) as *Proteus mirabilis*, and one (0.7%) as *Morganella morganii*. Regarding ESBL production, the double-disk synergy test (DDST) confirmed that 87.7% (128/146) of the isolates were ESBL producers. Notably, 95% of *E. coli* isolates exhibited ESBL production, along with all recovered *K. pneumoniae, P. mirabilis*, and *E. vulneris* isolates. In contrast, only 15.4% (2/13) of *Salmonella* spp. isolates were ESBL producers, while the single *M. morganii* isolate was non-ESBL-producing (Table 1).

**Table 1:** Distribution of Gram-negative isolates and ESBL production among recovered species.

**Bacterial species**	**No. of isolates (*n* = 146)**	**% of total isolates**	**No. of ESBL producers**	**% ESBL positive per species**
***Escherichia coli***	120	82.2%	114	95.0%
***Escherichia vulneris***	1	0.7%	1	100%
***Salmonella*** **spp**.	13	8.9%	2	15.4%
***Klebsiella pneumoniae***	9	6.2%	9	100%
***Proteus mirabilis***	2	1.4%	2	100%
***Morganella morganii***	1	0.7%	0	0%
**Total**	146	100%	128	87.7%

### 3.2 Phenotypic and genotypic characterization of the resistance pattern

**Figure 1:**
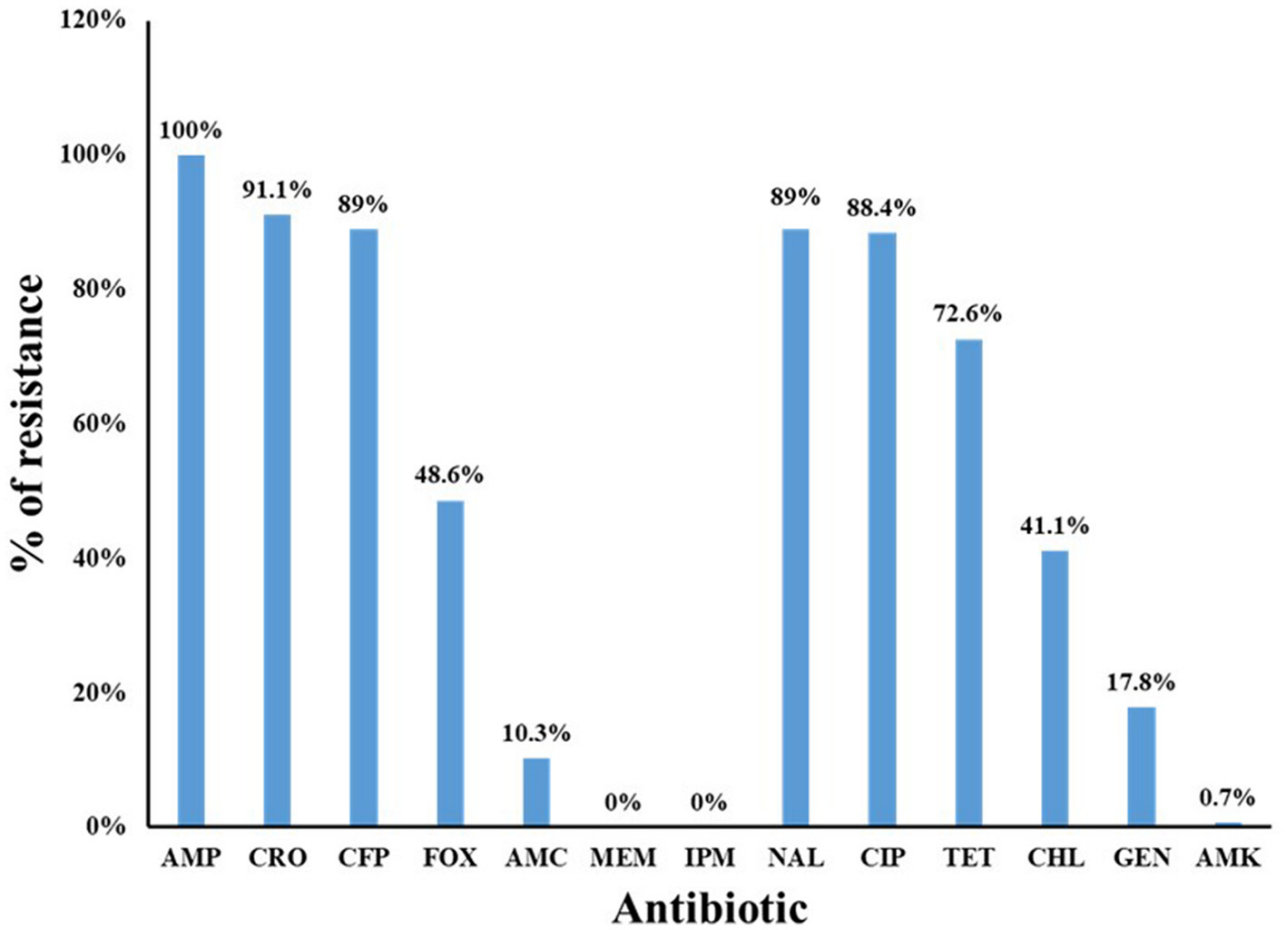
Antimicrobial resistance level for all studied Gram-negative strains (*n* = 146). The isolates showed high resistance rates to β-lactam antibiotics (except carbapenems), quinolones, tetracycline, and chloramphenicol with moderate to low resistance rates to aminoglycosides. Antibiotics: β-lactams [ampicillin (AMP), ceftriaxone (CRO), cefoperazone (CFP), cefoxitin (FOX), amoxicillin–clavulanic acid (AMC), meropenem (MEM), and imipenem (IPM)]; quinolones [nalidixic acid (NAL) and ciprofloxacin (CIP)]; tetracyclines [tetracycline (TET)]; phenicols [chloramphenicol (CHL)]; and aminoglycosides [gentamicin (GEN) and amikacin (AMK)].

Phenotypic antimicrobial susceptibility testing revealed high resistance rates to β-lactam antibiotics, ranging from 48.6% to 100%, except for AMC, which exhibited moderate resistance (10.3%), and carbapenems, to which no resistance was detected (Figure 1). Additionally, the isolates demonstrated high resistance to quinolones, with 89% resistant to NAL and 88.4% resistant to CIP. Resistance to TET and CHL was also notable, at 72.6% and 41.1%, respectively. In contrast, aminoglycoside resistance was relatively low, with 17.8% of isolates resistant to GEN and only 0.7% resistant to AMK (Figure 1).

**Figure 2:**
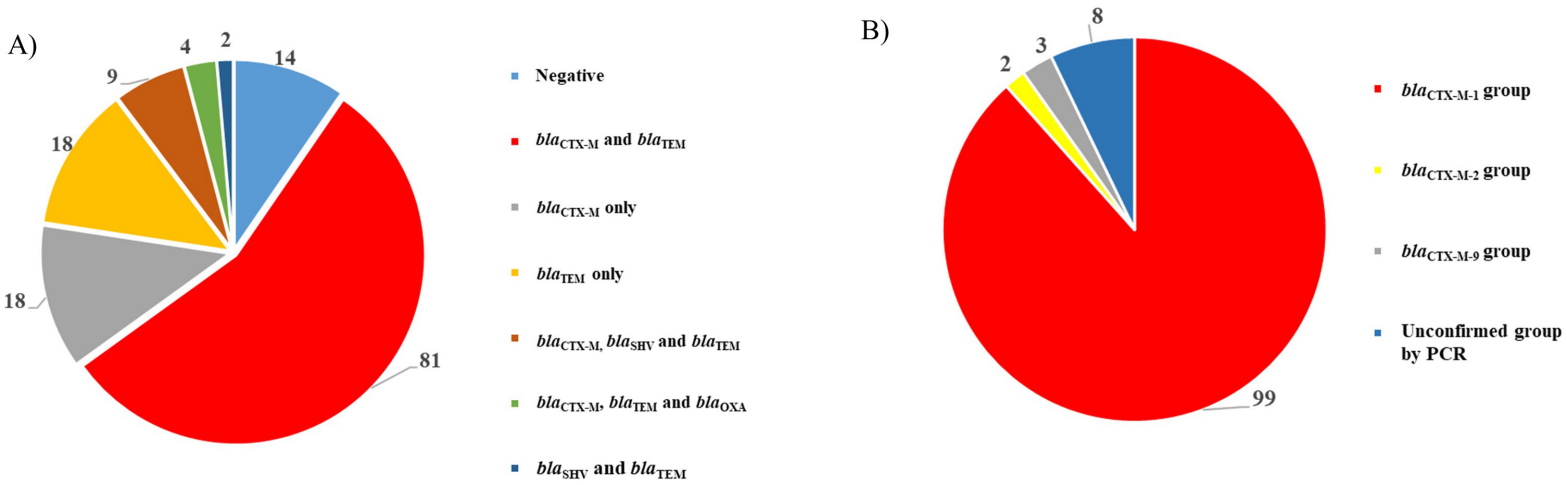
Genetic characterization of all the tested Gram-negative strains (*n* = 146). **(A)** represents the identified β-lactamases encoding genes. **(B)** represents the identified *bla*_CTX−M_ groups.

Genotypic analysis revealed the presence of β-lactam resistance genes among the isolates. The most frequently detected genes were *bla*_TEM_ and *bla*_CTX−M_, identified in 114 (78.1%) and 112 (76.7%) isolates, respectively. Additionally, *bla*_SHV_ and *bla*_OXA_ were detected in 11 (7.5%) and 4 (2.7%) isolates, respectively. Notably, multiple β-lactam resistance genes were co-harbored by several isolates, with *bla*_CTX−M_ and *bla*_TEM_ co-occurring in 81 isolates (55.5%), *bla*_CTX−M_, *bla*_SHV_, and *bla*_TEM_ identified in 9 isolates (6.2%), *bla*_CTX−M_, *bla*_TEM_, and *bla*_OXA_ in 4 isolates (2.7%), and *bla*_SHV_ and *bla*_TEM_ in 2 isolates (1.4%) (Figure 2A). Regarding *bla*_CTX−M_ classification, the *bla*_CTX−M−1_ group was the most prevalent, detected in 99 isolates (67.8%), followed by the *bla*_CTX−M−9_ group in 3 isolates (2.1%) and the *bla*_CTX−M−2_ group in 2 isolates (1.4%) (Figure 2B). Interestingly, 8 isolates initially tested positive for *bla*_CTX−M_ by PCR, but the specific group could not be determined. Whole-genome sequencing (WGS) later confirmed that all these isolates harbored *bla*_CTX−M−8_, a member of the *bla*_CTX−M−1_ group (Figure 2B). Additionally, PCR analysis identified the presence of the *mcr* gene in 15 isolates (10.3%). WGS further confirmed that all these isolates were colistin-resistant *E. coli* carrying the *mcr-1.1* gene.

### 3.3 Genetic characterization of selected isolates by WGS

WGS was conducted to fully characterize the selected 31 *E. coli* isolates. Among the *E. coli* isolates, the *bla*_CTX−M−1_ group was detected in 23 isolates, with *bla*_CTX−M−55_ being the most prevalent variant, identified in 10 isolates (43.5%), followed by *bla*_CTX−M−15_ in five isolates (21.7%) (Table 2). Notably, eight isolates initially tested positive for an unconfirmed *bla*_CTX−M_ group were later identified as *bla*_CTX−M−8_, highlighting the superior accuracy of WGS over PCR in determining genetic determinants. Additionally, two *E. coli* isolates carried *bla*_CTX−M−2_ group (*bla*_CTX−M−2_), while one isolate carried *bla*_CTX−M−9_ group (*bla*_CTX−M−14_). The *bla*_TEM_ gene was identified in 23 isolates, with 17 harboring non-ESBL variants (*bla*_TEM − 1B_ in nine isolates, *bla*_TEM − 176_ in five, *bla*_TEM − 1A_ in two, and *bla*_TEM − 99_ in one). Meanwhile, six isolates carried ESBL-producing *bla*_TEM_ variants (*bla*_TEM − 214_ four in isolates, *bla*_TEM − 215_ in one, and *bla*_TEM − 30_ in one). Additionally, *bla*_SHV − 12_ was detected in a single isolate. Interestingly, two isolates were phenotypically classified as ESBL producers but did not harbor any known ESBL-encoding genes. One of these isolates carried only *bla*_TEM − 176_, while the other harbored only *bla*_TEM − 1B_. Furthermore, the inclusion of two non-ESBL-producing *E. coli* isolates (S202C and S357C) in the WGS analysis aimed to validate the initial phenotypic resistance profiles and PCR screening results. Whole-genome sequencing confirmed the presence of non-ESBL *bla*_TEM_ variants, specifically *bla*_TEM − 1B_ in S202C and *bla*_TEM − 176_ in S357C (Table 2). These β-lactamase variants are not associated with extended-spectrum activity, supporting the classification of these isolates as non-ESBL producers. This finding highlights the consistency between phenotypic, molecular, and genomic data and underscores the importance of WGS in accurately characterizing resistance determinants, particularly in cases where phenotypic ambiguity may arise.

**Table 2:** Full genetic characterization of the selected 31 *E. coli* isolates based on whole genome sequencing.

**No**	**Isolate ID**	**Bacteria species (ST)**	**ESBL production**	**Resistant genes**	**Mutations**	**Plasmids**
1	S48C	*E. coli* (ST189)	Positive	*bla*_CTX−M−55_, *bla*_TEM − 214_, *mcr-1.1, aac(3)-Iid, aadA2, aadA22, aph(3″)-Ib, aph(6)-Id, dfrA12, catA1, erm(B), floR, fosA3, mph(A), qacE, tet(A), tet(M), sul1, sul2, sul3*	*gyrA* (D87N), *gyrA* (S83L), *parC* (S80I)	Col(BS512), IncHI2, IncHI2A, IncY
2	S64C1	*E. coli* (ST46)	Positive	*bla*_CTX−M−15_, *bla*_TEM − 30_, *mcr-1.1, aac(3)-Iid, ant(3″)-Ia, aph(3′)-Ia, aadA17, dfrA12, floR, cmlA1, qnrS1, tet(A), sul2, sul3*		IncI2(Delta), IncX1, p0111
3	S64C2	*E. coli* (ST57)	Positive	*bla*_CTX−M−2_, *bla*_TEM − 1A_, *aac(3)-Iia, ant(2″)-Ia, aph(3″)-Ib, aph(3′)-Ia, aph(6)-Id, aadA1, dfrA1, floR, cmlA1, tet(A), sul1, sul2, sul3*	*gyrA* (D87N), *gyrA* (S83L), *parC* (S80I)	IncFIB(AP001918), IncHI2, IncHI2A
4	S99C	*E. coli* (ST117)	Positive	*bla*_CTX−M−55_, *mcr-1.1, aac(3)-Iid, aph(3″)-Ib, aph(6)-Id, aadA1, aadA2, cmlA1, floR, qnrS1, sitABCD, tet(A), sul2, sul3*	*parC* (S80R)	ColpVC, IncFIB(AP001918), IncFII, IncI2(Delta)
5	S115C	*E. coli* (ST359)	Positive	*bla*_CTX−M−55_, *bla*_TEM − 214_, *mcr-1.1, aph(3″)-Ib, aph(3′)-Ia, aph(6)-Id, aadA1, aadA2, catA1, cmlA1, floR, sitABCD, tet(A), sul2, sul3*	*gyrA* (D87Y), *gyrA* (S83L), *parC* (S80I)	IncB/O/K/Z, IncFIB(AP001918), IncFII, IncFII(pHN7A8), IncI1-I(Alpha), IncI2(Delta), p0111
6	S131C	*E. coli* (ST117)	Positive	*bla*_CTX−M−55_, *mcr-1.1, aac(3)-Iid, aph(3″)-Ib, aph(6)-Id, aadA1, aadA2, cmlA1, floR, qnrS1, sitABCD, tet(A), sul2*	*parC* (S80R)	ColpVC, IncFIB(AP001918), IncFII, IncI2(Delta)
7	S136C1	*E. coli* (ST3489)	Positive	*bla*_CTX−M−14b_, *bla*_TEM − 1B_, *aac(3)-Iid, aac(3)-Via, aph(3′)-Ia, aph(6)-Id, dfrA14, floR, lnu(F), ARR-2, mph(A), qacE, qnrS1, sitABCD, tet(A), sul1, sul2*	*gyrA* (S83L)	IncFIB(AP001918), IncHI2, IncHI2A, IncI1-I(Alpha), IncI2, p0111
8	S136C2	*E. coli* (ST457)	Positive	*bla*_SHV − 12_, *bla*_TEM − 1B_, *mcr-1.1, aac(3)-Iid, aph(3″)-Ib, aph(3′)-Ia, aph(6)-Id, aadA1, aadA2, dfrA17, cmlA1, floR, qacE, sitABCD, tet(A), sul2, sul3*	*parC* (S80I), *parE* (S458A)	IncFIB(AP001918), IncX4
9	S165C	*E. coli* (ST162)	Positive	*bla*_CTX−M−15_, *bla*_TEM − 1B_*, mcr-1.1, aac(3)-Iia, aadA2, aadA24, aph(3″)-Ib, aph(3′)-Ia, aph(6)-Id, dfrA14, cmlA1, erm(B), mph(A), sitABCD, tet(A), sul3*	*parC* (S80I), *gyrA* (S83L), *gyrA* (D87N)	IncFIB(AP001918), IncI2(Delta), IncY
10	S166C1	*E. coli* (ST101)	Positive	*bla*_CTX−M−8_, *bla*_TEM − 1A_, *mcr-1.1, aac(3)-Iia, aadA1, aph(3″)-Ib, aph(3′)-Ia, aph(6)-Id, dfrA14, catA1, cmlA1, floR, mph(A), sitABCD, tet(A), sul2, sul3*	*gyrA* (D87Y), *gyrA* (S83L), *parC* (S80I)	IncFIB(AP001918), IncFIB(pLF82-PhagePlasmid, IncHI2, IncI1-I(Alpha)
11	S166C2	*E. coli* (ST354)	Positive	*bla*_CTX−M−55_, *bla*_TEM − 215_, *mcr-1.1, aadA1, dfrA14, fosA3, qnrS1, tet(A)*	*parC* (E84G), *parC* (S80I)	IncFII(pHN7A8), IncI(Gamma), IncI2(Delta)
12	S167C1	*E. coli* (ST10)	Positive	*bla*_CTX−M−55_, *bla*_TEM − 214_, *mcr-1.1, aac(3)-IV, aadA22, aph(4)-Ia, dfrA14, fosA3, lnu(F), qnrS1, tet(A)*		IncFIB(AP001918), IncFII(pHN7A8), IncI1-I(Alpha), IncI2(Delta), p0111
13	S167C2	*E. coli* (ST2172)	Positive	*bla*_CTX−M−15_, *bla*_TEM − 176_, *mcr-1.1, aadA2, ant(3″)-Ia, aph(3′)-Ia, cmlA1, floR, sitABCD, tet(A), sul3*	*gyrA* (D87N), *gyrA* (S83L), *parC* (S80I)	IncB/O/K/Z, IncFIA, IncFIB(AP001918), IncI1-I(Alpha), IncI2(Delta), IncX1, p0111
14	S199C	*E. coli* (ST694)	Positive	*bla*_TEM − 1B_, *aph(3″)-Ib, aph(6)-Id, dfrA14, fosA4, sitABCD, sul2*	*gyrA* (D87N), *gyrA* (S83L), *parC* (S80I)	IncFIA, IncFIA(HI1), IncFIB(AP001918), IncHI1A, IncHI1B(R27), IncI2
15	S200C1	*E. coli* (ST1771)	Positive	*bla*_CTX−M−15_, *mcr-1.1, aac(3)-Iia, aph(3″)-Ib, aph(3′)-Ia, aph(6)-Id, aadA2, aadA24, dfrA1, cmlA1, erm(B), floR, sitABCD, tet*(A), *sul3*	*gyrA* (S83L)	IncFIA, IncI2(Delta)
16	S200C2	*E. coli* (ST162)	Positive	*bla*_CTX−M−15_, *bla*_TEM − 1B_, *mcr-1.1, aac(3)-IIa, aadA2, aadA24, aph(3″)-Ib, aph(3′)-Ia, aph(6)-Id, dfrA14, cmlA1, erm(B), mph(A), sitABCD, tet(A), sul3*	*gyrA* (D87N), *gyrA* (S83L), *parC* (S80I)	IncFIB(AP001918), IncI2(Delta), IncY
17	S202C	*E. coli* (ST694)	Negative	*bla*_TEM − 1B_, *ant(3″)-Ia, aph(3″)-Ib, aph(6)-Id, dfrA14, fosA4, sitABCD, sul2, sul3*	*gyrA* (D87N), *gyrA* (S83L), *parC* (S80I)	IncFIA, IncFIA(HI1), IncFIB(AP001918), IncHI1A, IncHI1B(R27), IncI2
18	S203C	*E. coli* (ST694)	Positive	*bla*_CTX−M−8_, *bla*_TEM − 1B_, *aph(3″)-Ib, ant(3″)-Ia, aph(6)-Id, dfrA14, fosA4, sitABCD, sul2, sul3*	*gyrA* (D87N), *gyrA* (S83L), *parC* (S80I)	IncFIA, IncFIA(HI1), IncFIB(AP001918), IncHI1A, IncHI1B(R27), IncI2
19	S259C	*E. coli* (ST694)	Positive	*bla*_CTX−M−8_, *bla*_TEM − 1B_, *ant(3″)-Ia, aph(3″)-Ib, aph(6)-Id, dfrA14, fosA4, sitABCD, sul2, sul3*	*gyrA* (D87N), *gyrA* (S83L), *parC* (S80I)	IncFIA, IncFIA(HI1), IncFIB(AP001918), IncHI1A, IncHI1B(R27), IncI1-I(Alpha), IncX4
20	S261C1	*E. coli* (ST195)	Positive	*bla*_CTX−M−55_, *bla*_TEM − 176_, *mcr-1.1, aph(3″)-Ib, aph(3′)-Ia, aph(6)-Id, aadA1, aadA2, cmlA1, fosA3, qnrS13, sul3*	*gyrA* (D87N), *gyrA* (S83L), *parC* (S80I)	ColE10, IncI(Gamma), IncX1, IncX4, p0111
21	S279C	*E. coli* (ST10)	Positive	*bla*_CTX−M−8_, *sitABCD*		IncFIA, IncFIB(AP001918), IncI1-I(Alpha)
22	S296C	*E. coli* (ST10)	Positive	*bla*_TEM − 176_, *ant(3″)-Ia, aph(3′)-Ia, mcr-1.1, fosA4, qnrS1, sul3*	*gyrA* (D87N), *gyrA* (S83L), *parC* (S80I)	IncI1-I(Alpha), IncI2, IncX1, IncX4, p0111
23	S316C	*E. coli* (ST694)	Positive	*bla*_CTX−M−8_, *bla*_TEM − 176_, *ant(3″)-Ia, aph(3′)-Ia, fosA4, qnrS1, sul3*	*gyrA* (D87N), *gyrA* (S83L), *parC* (S80I)	IncFIA, IncFIA(HI1), IncFIB(AP001918), IncHI1A, IncHI1B(R27), IncI1-I(Alpha), IncX1
24	S314C1	*E. coli* (ST155)	Positive	*bla*_CTX−M−8_, *bla*_CMY − 2_*, sitABCD, tet(A)*	*gyrA* (D87N), *gyrA* (S83L), *parC* (S80I)	IncFIB(AP001918), IncI1-I(Alpha), IncX4
25	S314C2	*E. coli* (ST1011)	Positive	*bla*_CTX−M−8_, *bla*_TEM − 1B_, *aac(3)-Iid, mph(A), sitABCD, tet(A)*	*parC* (S80I)	IncFIB(AP001918), IncI1-I(Alpha), IncQ1
26	S335C2	*E. coli* (ST215)	Positive	*bla*_CTX−M−55_, *bla*_TEM − 99_, *aph(3′)-Ia, fosA3, qnrS1, sitABCD, tet(A)*	*gyrA* (D87N), *gyrA* (S83L), *parC* (S80I)	IncB/O/K/Z, IncFIA, IncFIB(AP001918), IncFII(pHN7A8), IncN, IncX1
27	S336C	*E. coli* (ST6751)	Positive	*bla*_CTX−M−55_, *bla*_TEM − 214_, *aph(3′)-Ia, fosA3, qnrS1, tet(A)*	*gyrA* (D87N), *gyrA* (S83L), *parC* (S80I)	IncFII(pHN7A8), IncN, IncX1, p0111
28	S357C	*E. coli* (ST694)	Negative	*bla*_TEM − 176_, *ant(3″)-Ia, aph(3′)-Ia, fosA4, qnrS1, sitABCD, sul3*	*gyrA* (D87N), *gyrA* (S83L), *parC* (S80I)	IncFIA, IncFIA(HI1), IncFIB(AP001918), IncHI1A, IncHI1B(R27), IncI1-I(Alpha), IncX1
29	S358C	*E. coli* (ST155	Positive	*bla*_CTX−M−8_, *sitABCD, tet(A)*	*gyrA* (D87N), *gyrA* (S83L), *parC* (S80I)	IncFIA, IncFIB(AP001918), IncI1-I(Alpha), IncI2
30	S367C	*E. coli* (ST57)	Positive	*bla*_CTX−M−2_, *ant(2″)-Ia, dfrA1, qacE, sitABCD, tet(A), sul1, sul2*	*gyrA* (D87N), *gyrA* (S83L), *parC* (S80I)	IncFIB(AP001918), IncY, aadA1
31	S377C	*E. coli* (ST155)	Positive	*bla*_CTX−M−55_, *aac(3)-Iia, aph(3′)-Ia, fosA4, sitABCD, tet(A)*	*gyrA* (D87N), *gyrA* (S83L), *parC* (S80I)	IncB/O/K/Z, IncFIA(HI1), IncFIB(AP001918), IncFIB(pLF82-PhagePlasmid), IncHI1A, IncHI1B(R27)

In addition to ESBL genes, the *E. coli* isolates harbored a diverse range of antimicrobial resistance (AMR) determinants (Table 2, Supplementary Table S3). Aminoglycoside resistance genes were found in 28 isolates, with *aph(3*′*)-Ia, aph(6)-Id*, and *aph(3*″*)-Ib* detected in 17, 16, and 15 isolates, respectively. Sulfonamide genes occurred in 23 isolates, mainly *sul3* (18 isolates). Tetracycline resistance was observed in 22 isolates, all carrying *tet(A)*. Trimethoprim genes appeared in 16 isolates, dominated by *dfrA14* (10 isolates). Colistin resistance was limited to *mcr-1.1* in 15 isolates. Fosfomycin genes were identified in 14 isolates (*fosA4*, 8; *fosA3*, 6). Phenicols resistance involved *cmlA1* (12) and *floR* (11) in 14 isolates. Quinolone resistance genes were found in 12 isolates, mainly *qnrS1* (11). Macrolide genes occurred in seven isolates—three carrying both *mph(A)* and *erm(B)*, three only *mph(A)*, and one only *erm(B)*. Lincosamide (*lnu(F)*) and rifampicin (*arr-2*) resistance appeared in two and one isolates, respectively. Finally, disinfectant resistance genes were detected in 23 isolates, with *sitABCD* being most common (22 isolates).

Notably, 29 out of the 31 *E. coli* tested isolates carried at least one mutation linked to quinolone resistance (Table 2). Among the detected mutations, *parC* (S80I) and *gyrA* (S83L) were the most prevalent, found in 24 and 23 isolates, respectively. Additionally, other mutations were identified, including *gyrA* (D87N) in 19 isolates, *gyrA* (D87Y) and *parC* (S80R) in two isolates each, and *parC* (E84G) and *parE* (S458A) in one isolate each.

**Figure 3:**
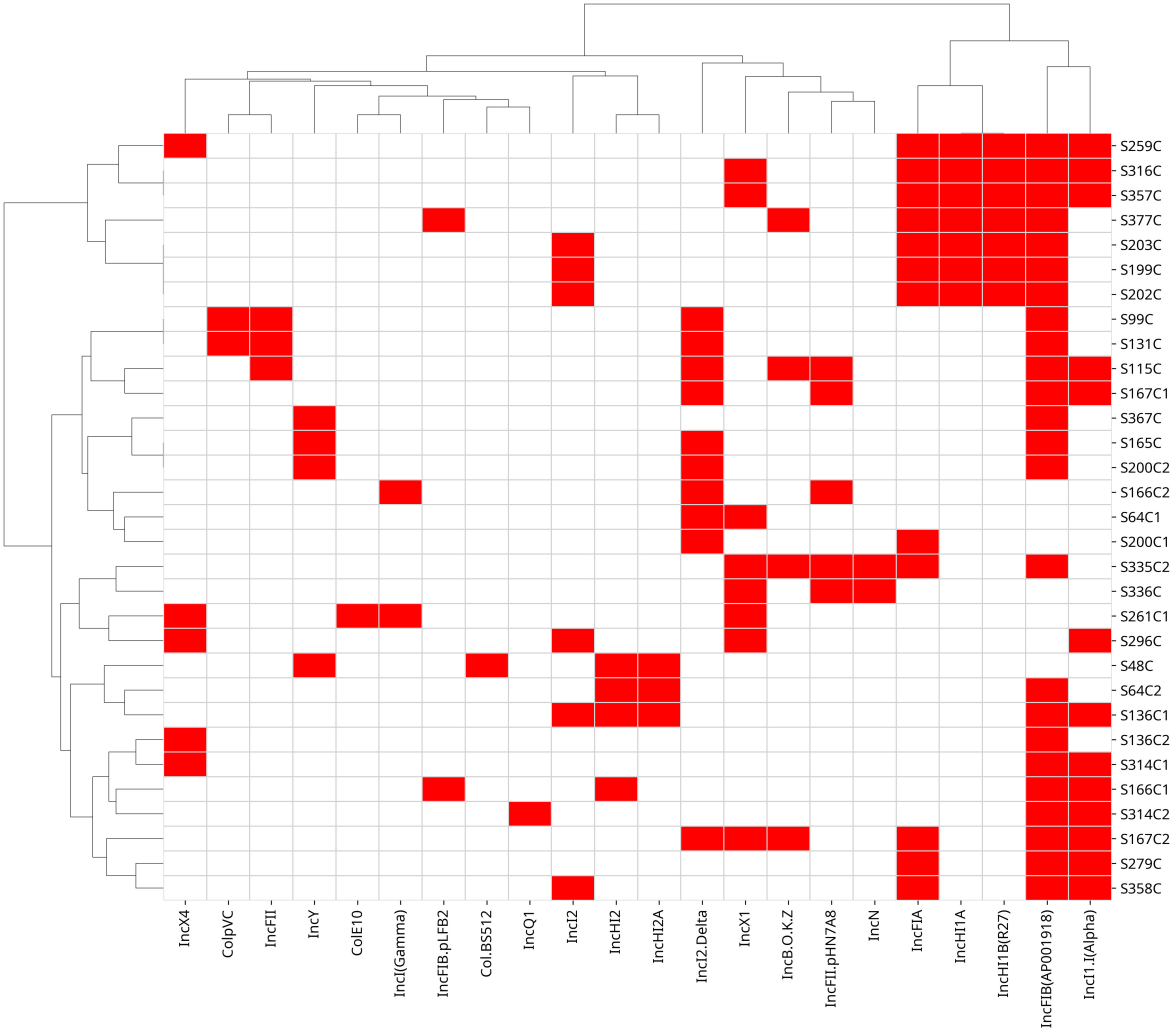
Distribution of plasmid replicons in *E. coli* isolates. Red markings indicate the presence of a resistance gene, while white markings denote its absence. The samples are arranged by isolate ID.

All *E. coli* isolates carried at least one plasmid replicon type (Table 2, Figure 3). IncF plasmids were most prevalent, particularly IncFIB(AP001918) (24 isolates), followed by IncFIA (11 isolates), IncFIA(HI1) (7), IncFII(pHN7A8) (5), IncFII (3), and IncFIB(pLF82-PhagePlasmid) (2). IncI plasmids ranked second, including IncI1-I(Alpha) (13 isolates), IncI2(Delta) (10), IncI2 (6), and IncI(Gamma) (2). IncH plasmids were also frequent, such as IncHI1A and IncHI1B(R27) (7 isolates each), IncHI2 (4), and IncHI2A (3). Other replicons, including IncX1, IncY, and ColpVC, were detected in smaller numbers.

In our study, a variety of virulence-associated genes relevant to *E. coli* were identified (Supplementary Figure S1). Among these, the *iroN, iroC*, and *iroE* genes—part of the *iroA* gene cluster involved in siderophore-mediated iron acquisition—were the most prevalent, detected in 19 isolates. These genes are commonly associated with extraintestinal pathogenic *E. coli* (ExPEC). Other frequently identified genes included *yagZ/ecpA* (*n* = 18), *fimE* (*n* = 12), and *yagW/ecpD* (*n* = 10), all of which are involved in adhesion and biofilm formation. Additional ExPEC-related adhesion factors such as *fimC, fimD, fimI, papB*, and members of the *csg* operon (*csgB–G*) were also observed. Iron uptake systems including *chuU, chuV, fes, fyuA, irp1, irp2*, and components of the *ybt* operon were present in several isolates, supporting the potential for enhanced pathogenic fitness. Toxins such as *astA, cdtB, pic*, and *vat*, commonly associated with diarrheagenic and uropathogenic *E. coli*, were also detected at varying frequencies. Importantly, we did not identify key hallmark genes associated with enterohemorrhagic (EHEC, e.g., *stx1, stx2*), enteropathogenic (EPEC, e.g., *eae, bfpA*), or enterotoxigenic (ETEC, e.g., *elt, est*) *E. coli*, suggesting the isolates primarily harbor virulence traits typical of ExPEC.

### 3.4 *E. coli* sequence typing and phylogenetic relationship of the isolates

WGS-based sequence typing of *E. coli* isolates identified 19 distinct STs (Table 2). Our findings showed that ST694 was the most prevalent, appearing in five isolates recovered from the cecal contents and fecal samples of different farms within the same poultry farm (Supplementary Table S4). ST10 and ST155 were each identified in three isolates, with ST10 isolates coming from two different poultry farms and ST155 isolates from the same farm. Additionally, ST117, ST162, ST195, and ST2172 were identified in two isolates each. Notably, ST162 isolates were from two different poultry farms, while ST117, ST195, and ST2172 isolates were from the same poultry farm (Supplementary Table S4).

**Figure 4:**
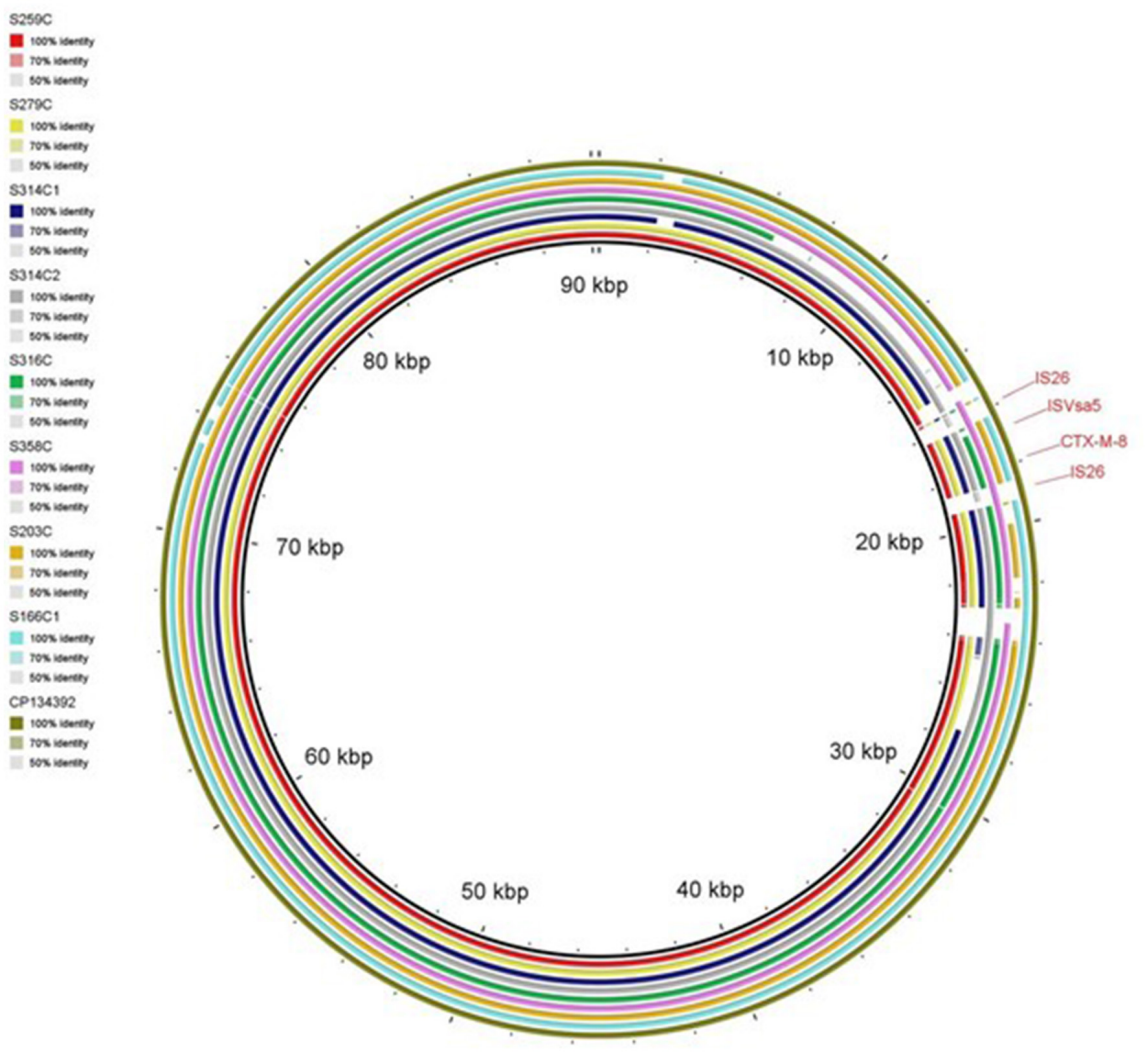
Circular map illustrating the reconstructed plasmids from the WGS of *E. coli* isolates carrying *bla*_CTX−M−8_ gene compared to retrieved plasmid sequences from NCBI. The plasmids were displayed from inner to outer circles in the following order: S259C, S279C, S314C1, S314C2, S316C, S358C, S203C and S166C1. The out-layer circle (olive green color) represents the reference plasmid pMTY10828_InCI1-I (accession no. CP134392.1) used for sequence comparison. The figure was generated using BLAST Ring Image Generator (BRIG) tool (http://sourceforge.net/projects/brig).

WGS analysis identified plasmids harboring the *bla*_CTX−M−8_ gene in *E. coli* isolates, with sequence comparisons conducted against the reference IncI1 plasmid pMTY10828_InCI1-I (CP134392.1). The circular map, generated using the BLAST Ring Image Generator (BRIG), revealed a high degree of sequence similarity between the reconstructed plasmids (S259C, S279C, S314C1, S314C2, S316C, S358C, S203C, and S166C1) and the reference plasmid (Figure 4). Most of the plasmids shared conserved backbone regions characteristic of IncI1-type plasmids, while exhibiting variability in specific segments likely associated with mobile genetic elements and resistance determinants. The strong homology with the IncI1 reference plasmid suggests its potential role as a key vector in the dissemination of the *bla*_CTX−M−8_ gene among diverse *E. coli* strains.

**Figure 5:**
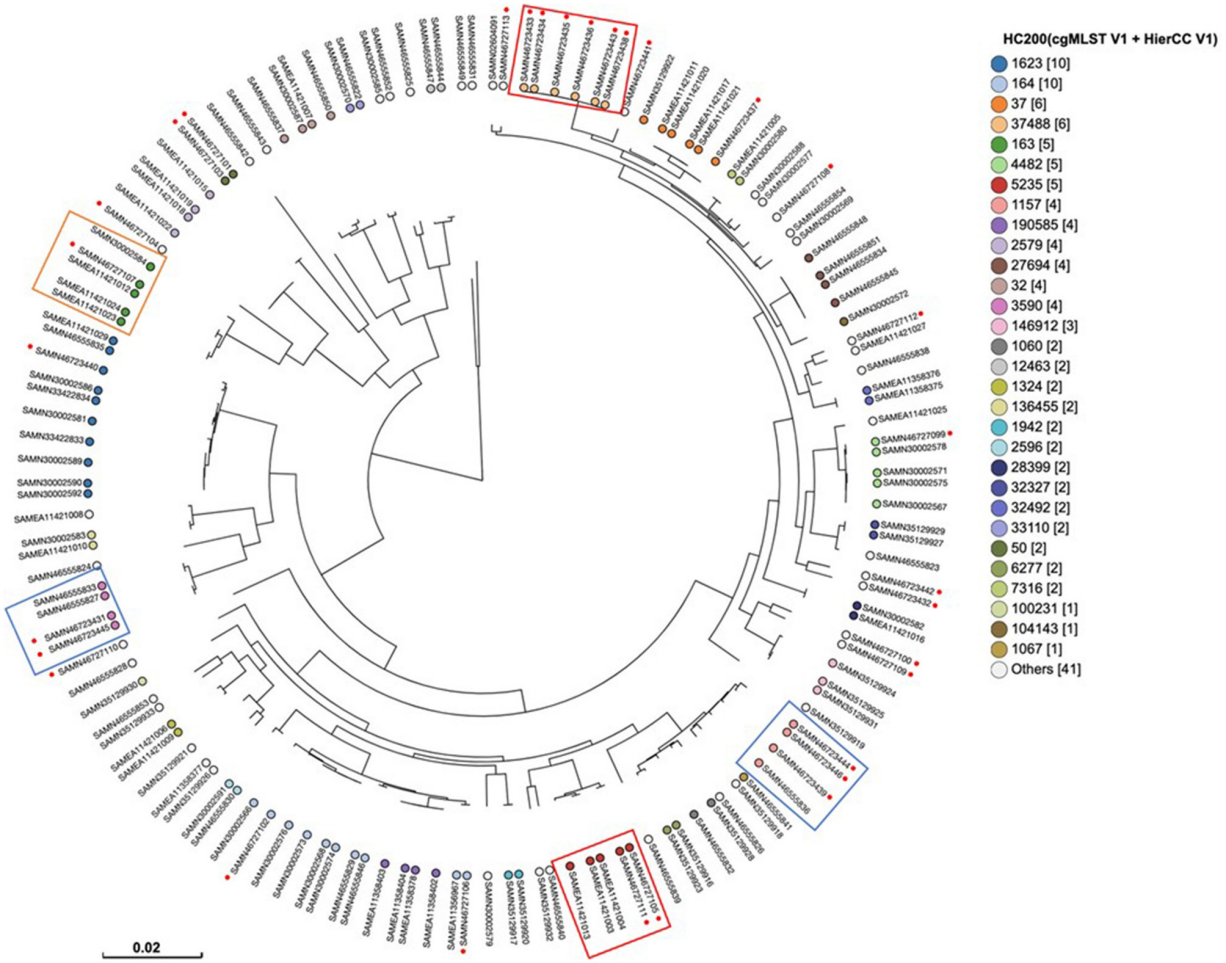
SNPs and hierarchical clustering of cgMLST (HierCC) of the examined *E. coli* isolates (highlighted with a red dot) with 112 publicly available *E. coli* sequences from the UAE, representing diverse sources in EnteroBase (https://enterobase.warwick.ac.uk/species/index/ecoli). Tip labels indicate the HC200 cgMLST pattern, defined by ≤200 allelic differences. Isolates recovered in this study are indicated by red dots. Isolates exhibiting close genetic relatedness to human-derived strains are highlighted with red boxes, those showing similarity to both human and environmental isolates are marked with orange boxes, and isolates closely related to broiler carcass strains are enclosed in blue boxes.

Phylogenetic analysis based on single nucleotide polymorphisms (SNPs) and hierarchical clustering of core-genome multilocus sequence typing (cgMLST) was performed to assess the epidemiological relatedness between the study isolates and 112 publicly available *Escherichia coli* genomes from the UAE, derived from diverse sources (Figure 5). The isolates were classified into 31 distinct clusters at the HC50 level, which groups isolates differing by no more than 200 alleles across 2,513 core genomic loci. The most frequently observed HC50 clusters were HC50|163 and HC50|164, each comprising 10 isolates, followed by HC50|37 and HC50|37488, each including 6 isolates. Among our isolates, HC50|37488 was the most predominant (*n* = 6), followed by HC50|1157 (*n* = 3). Most importantly, several of our isolates clustered with *E. coli* strains from human and broiler carcass sources that shared identical HC50 patterns, indicating potential human cross transmission. For instance, isolate S279C (SAMN46723437; ST10) clustered within HC50|37 alongside four human isolates (SAMEA11421011, SAMEA11421017, SAMEA11421020, and SAMEA11421021) belonging to ST1585. Similarly, two isolates (SAMN46727105 and SAMN46727111; ST162) grouped within HC50|5235 with three human isolates (SAMEA11421003 and SAMEA11421004, ST12220; SAMEA11421013, ST162). Isolate SAMN46727107 (ST354) clustered in HC50|163 with three human isolates (SAMEA11421012, SAMEA11421023, and SAMEA11421024) and one environmental isolate (SAMN30002584), all sharing ST354. Additionally, three isolates (SAMN46723439, SAMN46723444, and SAMN46723446; ST115) were classified within HC50|1157 alongside a broiler carcass isolate (SAMN46555836; ST155). Furthermore, two isolates (SAMN46723445 and SAMN46723431; ST57) shared the HC50|3590 cluster with broiler carcass isolates SAMN46555827 and SAMN46555833 (ST4753). These findings underscore the genetic similarity between our isolates and publicly available strains from both human and food chain sources, suggesting possible transmission events and highlighting the importance of integrated One Health surveillance.

## 4 Discussion

The rising prevalence of ESBL-producing Gram-negative bacteria in poultry presents a serious challenge to animal health, food safety, and public health. In the UAE, where poultry farming plays a vital role in food security, the emergence of antimicrobial-resistant pathogens can lead to economic losses due to reduced productivity, increased disease outbreaks, and the potential transmission of resistant bacteria to humans through the food chain. Understanding the genetic basis of ESBL resistance in chicken-associated bacterial isolates is crucial for developing effective mitigation strategies, enhancing biosecurity measures, and guiding antibiotic stewardship policies. Our study revealed a high prevalence of ESBL-producing Gram-negative bacteria in the UAE (87.7%), with *E. coli* being the dominant species. It is important to note that this study assessed prevalence in two major poultry companies, which do not represent the entire UAE poultry industry. Therefore, larger-scale studies are needed for a more comprehensive analysis. Similar to our finding, high prevalence rates of ESBL-producing *E. coli* have been reported in neighboring countries, such as Egypt (75%) (26), and in other regions, including Malaysia (45.4%) (27). In contrast, moderate prevalence levels have been observed in Saudi Arabia, with 34.7% in broilers and 19.1% in backyard chickens (28), while a notably lower prevalence (2.2%) of ESBL-producing *E. coli* was reported in commensal isolates from fecal samples in Qatar (29). Although this is the first study to evaluate the prevalence and genetic basis of ESBL production in chicken cecal contents and cecal drops in the UAE, particularly in *E. coli*, previous research has documented high levels of ESBL-producing *E. coli* in chicken products. For instance, our earlier study found that 79.68% of tested supermarket chicken meat contained ESBL-producing *E. coli* (11), emphasizing the urgent need to implement strategies to control the emergence of ESBL-producing *E. coli* in the UAE poultry industry. For isolates exhibiting an ESBL-positive phenotype but lacking detectable ESBL-encoding genes, further studies are warranted to elucidate the underlying mechanisms. Future investigations should include promoter region sequencing to identify possible regulatory mutations and exploration of non-enzymatic resistance mechanisms, such as changes in outer membrane permeability or efflux pump activity, which may contribute to the observed phenotypic resistance.

In addition to the high prevalence of ESBL production observed in our study, the isolates also exhibited alarming resistance levels to several critically important antimicrobials, including quinolones, tetracyclines, chloramphenicol, and colistin. Previous research on ESBL-producing *E. coli* from supermarket chicken meat in the UAE reported similarly high resistance rates to clinically significant antimicrobials, particularly ciprofloxacin (80%), cefepime (46%), and colistin (7%) (11), which aligns with our findings. A similar trend of increased antimicrobial resistance among ESBL-producing isolates from poultry has also been documented in other countries, including Egypt (26), Saudi Arabia (28), and Malaysia (27). Notably, quinolones, tetracyclines, β-lactams, polymyxins, and sulfonamides are among the most commonly used antimicrobials in poultry production (30). Consequently, the high resistance levels observed pose a significant concern, as they limit therapeutic options and facilitate the transmission of resistance genes to humans, potentially leading to untreatable infections.

Genetic analysis, including PCR and WGS of selected isolates, confirmed that ESBL production is primarily attributed to the *bla*_CTX−M_ gene, specifically group 1. Our findings partially align with a previous study from the Netherlands, which reported *bla*_CTX−M−1_–a member of *bla*_CTX−M−1_ group—as the predominant ESBL genotype in chicken meat (31). In contrast, a study from Egypt identified *bla*_CTX−M−9_ group as the most prevalent (26). Through WGS, we determined that *bla*_CTX−M−55_ is the most common variant within Group 1, which is consistent with our previous study on ESBL-producing *E. coli* from chicken carcasses in the UAE (11). These results underscore the significant role of this gene in the spread of ESBL production within the UAE poultry industry. Supporting our findings, previous studies have also reported a high prevalence of *bla*_CTX−M−55_ in broiler chicken farms in Brazil (32), and in raw retail chicken in South Korea (33). Furthermore, we identified the presence of *bla*_CTX−M−8_, another ESBL-associated gene, which has previously been reported in UAE chicken carcasses (11). The identification of *bla*_CTX−M−8_ in our study is important, as previous reports suggest the potential for local transmission of *bla*_CTX−M−8_ between humans and chickens via genetically diverse *E. coli* strains, contributing to the spread of antimicrobial resistance (34). The identification of *bla*_CTX−M−8_ within IncI1-type plasmids supports the previous finding and holds particular concern, as these plasmids have been widely implicated in the horizontal dissemination of ESBL genes across different bacterial populations and ecological niches (34). The high sequence similarity observed between the reconstructed IncI1 plasmids in this study and a reference IncI1 plasmid carrying *bla*_CTX−M−8_ (pMTY10828_InCI1-I; CP134392.1) further supports the role of this plasmid backbone in facilitating the regional or possibly global spread of this resistance gene.

The identification of diverse AMR genes and mutations in *E. coli* isolates from chickens in the UAE underscores the growing threat of MDR in poultry-associated bacteria. Beyond ESBL-encoding genes, our study revealed the presence of resistance determinants against multiple antibiotic classes, including aminoglycosides, sulfonamides, tetracyclines, trimethoprim, colistin, fosfomycin, phenicols, quinolones, macrolides, lincosamides, and rifampicin. The high prevalence of *aph(3*′*)-Ia, aph(6)-Id*, and *aph(3*″*)-Ib* among aminoglycoside resistance genes, as well as *sul3* in sulfonamide-resistant isolates, aligns with reports highlighting the widespread distribution of these resistance markers in poultry worldwide (35). Similarly, the near-universal presence of *tet(A)* in tetracycline-resistant isolates reflects the extensive use of tetracyclines in poultry farming, a common driver of resistance selection (36). A particularly concerning finding in our study was that 10.3% of the isolates carried the *mcr-1.1* gene, all of which were *E. coli* and colistin resistant. The implications of this colistin resistance will be further analyzed in future research.

Additionally, our findings revealed widespread quinolone resistance, with *qnrS1* being the most prevalent plasmid-mediated quinolone resistance gene, further supported by chromosomal mutations affecting the quinolone resistance-determining region of *parC, gyrA*, and *parE*. Notably, the *parC* (S80I) and *gyrA* (S83L) mutations were identified in over 70% of the tested isolates, which is consistent with previous studies linking these mutations to high-level fluoroquinolone resistance in avian *E. coli* strains (37). The presence of additional mutations, such as *gyrA* (D87N) and *parC* (S80R), further highlights the complexity of fluoroquinolone resistance mechanisms in poultry-associated bacteria. The detection of disinfectant resistance genes, particularly *sitABCD*, in 22 isolates suggests that selective pressure from farm disinfectants may contribute to the persistence and dissemination of resistant strains (38).

The identification of a diverse range of plasmid replicons and virulence-associated genes in *E. coli* isolates from chickens in the UAE highlights the potential for enhanced pathogenicity and AMR dissemination in poultry environments. Plasmids play a critical role in the horizontal transfer of AMR and virulence determinants, contributing to the emergence of MDR bacterial strains (39). Our study revealed that all isolates carried at least one plasmid replicon, with IncF plasmids being the most prevalent. Both IncF and IncI plasmids are well-recognized platforms for the horizontal transfer of multidrug resistance genes within the *Enterobacteriaceae* family, facilitating the dissemination of antimicrobial resistance among bacterial populations. The predominance of these plasmid families in our isolates provides strong evidence for the existence of an active genetic platform promoting the spread of resistance determinants in local poultry populations (40). Additionally, the identification of IncI, IncH, IncX, and IncY plasmids further underscores the genetic diversity of these isolates, as these plasmid types are commonly associated with virulence factors and resistance genes in pathogenic *E. coli* strains (41).

In addition to plasmid replicons, our findings revealed a broad spectrum of virulence-associated genes, particularly those involved in iron acquisition, adhesion, toxin production, and invasion. The iroA gene cluster (*iroN, iroC, iroE*), which was the most prevalent in our study, is enables bacteria to bypass this aspect of the innate immune defense, restoring their Ent-dependent iron uptake system and contributing significantly to their pathogenicity (42). The presence of adhesion-related genes such as *ecpA* and *fimE* suggests an enhanced ability of these isolates to adhere to host tissues, which is a key step in bacterial pathogenesis (43). Additionally, the identification of a wide range of type III secretion system genes further indicates the potential for these isolates to invade host cells and evade immune responses, thereby increasing their pathogenicity (44).

Interestingly, phylogenomic analysis confirmed a close genetic relationship between several of our *E. coli* isolates and strains previously recovered from two major sources in the UAE—humans and chicken carcasses—as well as with certain environmental isolates. This suggests potential cross-species and human transmission pathways. Supporting our observations, previous studies have reported a high degree of genetic similarity among *E. coli* strains from diverse sources, indicating complex bidirectional transmission dynamics involving chickens, wild animals, and the environment within poultry farming systems (45). Several isolates in our study were found to be closely related to human-associated *E. coli* strains and belonged to sequence types (STs) previously reported in human infections, underscoring their zoonotic relevance. Whole-genome sequencing (WGS) identified ST694 as the most prevalent sequence type in our dataset. This ST has previously been associated with zoonotic potential in neonatal calves in Uruguay (46). The next most common sequence types were ST10 and ST155, both well-documented in the context of zoonotic transmission (47, 48). Notably, ESBL-producing *E. coli* ST10 has been isolated from a wide range of sources, including chicken meat, other animal meats, rectal swabs from healthy individuals, and human blood cultures (48). This aligns with our findings, particularly the detection of several strains closely related to those from chicken carcasses. Similarly, ST155 has been recognized as a significant vector in the animal-to-human transmission of ESBL genes (47). Taken together, these results emphasize the urgent need for integrated surveillance and enhanced biosecurity practices in poultry farms to mitigate the zoonotic spread of multidrug-resistant ESBL-producing *E. coli*. Additionally, the observed genetic relatedness among isolates from different farms, as well as within individual farms (Supplementary Table S4), highlights potential intra- and inter-farm transmission pathways, further reinforcing the necessity for robust infection control strategies.

It is important to take into account the limitations of this study when interpreting the findings. The relatively small number of sequenced isolates may not fully reflect the genetic variation of ESBL-producing strains circulating in poultry throughout the UAE, despite the fact that the 31 *E. coli* isolates chosen for WGS were chosen to reflect the range of phenotypic and genetic diversity observed. Furthermore, this study only offers a snapshot of the current state of antimicrobial resistance because it was cross-sectional. Although the results do not offer concrete proof of such occurrences, they do raise the possibility of connections and transmission between farms, animals, and people. Finally, while the genomic analyses identified several plasmid types and virulence genes, we did not carry out laboratory-based validation such as conjugation tests or infection models. Future work combining genomic and functional studies will be important to confirm the activity of these genes and to better understand how resistance spreads among poultry-associated *E. coli* in the region.

## 5 Conclusion

In conclusion, this study confirmed a high prevalence of ESBL production among *E. coli* isolates recovered from chicken cecal contents and cecal droppings from two major producers in the UAE poultry sector. The isolates exhibited high levels of resistance to multiple critically important antimicrobials and carried a diverse array of antimicrobial resistance genes, virulence genes, and plasmid replicon types. ESBL production was primarily attributed to the *bla*_CTX−M−1_ group, with *bla*_CTX−M−55_ being the most prevalent among the tested isolates. Sequence typing and phylogenomic analysis revealed a close genetic relationship between several *E. coli* isolates and strains previously recovered from humans, chicken carcasses, and environmental sources in the UAE. These findings strongly suggest the involvement of poultry production systems in complex cross-species and zoonotic transmission pathways. The detection of sequence types commonly associated with human infections (e.g., ST10 and ST155) further underscores the zoonotic potential of these isolates. Additionally, the genetic relatedness observed among isolates from different farms and within individual farmhouses points to both intra- and inter-farm transmission, highlighting the role of the poultry environment as a reservoir and conduit for the spread of multidrug-resistant *E. coli*. Implementing strict hygiene measures, enhanced surveillance, and robust biosecurity programs is crucial to preventing the spread of these resistant pathogens in the UAE.

## Data Availability

The whole-genome sequence reads generated in this study have been submitted to GenBank under the BioProject ID numbers PRJNA1220707 (BioSample numbers SAMN46723431-SAMN46723446) and PRJNA1221085 (Biosample numbers SAMN46727099-SAMN46727113).

## References

[1] O'Neill J. Antimicrobial Resistance: Tackling a Crisis for the Health and Wealth of Nations. London: The Review on Antimicrobial Resistance (2016).

[2] Murray CJL Ikuta KS Sharara F Swetschinski L Aguilar GR Gray A . Global burden of bacterial antimicrobial resistance in 2019: a systematic analysis. Lancet. (2022) 399:629–55. doi: 10.1016/S0140-6736(21)02724-0 35065702 PMC8841637

[3] van Hoek AH Dierikx C Bosch T Schouls L van Duijkeren E. Transmission of ESBL-producing *Escherichia coli* between broilers and humans on broiler farms. J Antimicrob Chemother. (2020) 75:543–9. doi: 10.1093/jac/dkz507 31800052

[4] Khalifa HO Oreiby AF Abd El-Hafeez AA Abd El Latif A Okanda T Kato Y . High β-lactam and quinolone resistance of *Enterobacteriaceae* from the respiratory tract of sheep and goats with respiratory disease. Animals. (2021) 11:2258. doi: 10.3390/ani11082258 34438714 PMC8388476

[5] Khalifa HO Oreiby AF Okanda T Kato Y Matsumoto T. High β-lactam resistance in Gram-negative bacteria associated with kennel cough and cat flu in Egypt. Sci Rep. (2021) 11:3347. doi: 10.1038/s41598-021-82061-2 33558604 PMC7870956

[6] Ahmed AM Maruyama A Khalifa HO Shimamoto T. Seafood as a reservoir of Gram-negative bacteria carrying integrons and antimicrobial resistance genes in Japan. Biomed Environ Sci. (2015) 28:924–6. doi: 10.3967/bes2015.128 26777914

[7] Elbediwi M Tang Y. Genomic characterization of ESBL-producing *Salmonella Thompson* isolates harboring *mcr-9* from dead chick embryos in China. Vet Microbiol. (2023) 278:109634. doi: 10.1016/j.vetmic.2022.109634 36610099

[8] World Health Organization. Critically Important Antimicrobials for Human Medicine. 6th edn. Geneva: World Health Organization (2018). Available at: https://apps.who.int/iris/bitstream/handle/10665/312266/9789241515528-eng.pdf

[9] Cameron-Veas K Solà-Ginés M Moreno MA Fraile L. Impact of the use of β-lactam antimicrobials on the emergence of *Escherichia coli* isolates resistant to cephalosporins under standard pig-rearing conditions. Appl Environ Microbiol. (2015) 81:1782–7. doi: 10.1128/aem.03916-14 25548055 PMC4325139

[10] Habib I Elbediwi M Mohamed MYI Ghazawi A Abdalla A Khalifa HO . Enumeration, antimicrobial resistance and genomic characterization of extended-spectrum β-lactamases producing *Escherichia coli* from supermarket chicken meat in the United Arab Emirates. Int J Food Microbiol. (2023) 398:110224. doi: 10.1016/j.ijfoodmicro.2023.110224 37167788

[11] Habib I Elbediwi M Mohteshamuddin K Mohamed MY Lakshmi GB Abdalla A . Genomic profiling of extended-spectrum β-lactamase-producing *Escherichia coli* from pets in the United Arab Emirates: unveiling colistin resistance mediated by *mcr-1.1* and its probable transmission from chicken meat–A One Health perspective. J Infect Public Health. (2023) 16:163–171. doi: 10.1016/j.jiph.2023.10.034 37957104

[12] Lemlem M Aklilu E Mohamed M Kamaruzzaman NF Devan SS Lawal H . Prevalence and molecular characterization of ESBL-producing *Escherichia coli* isolated from broiler chicken and their respective farm environments in Malaysia. BMC Microbiol. (2024) 24:499. doi: 10.1186/s12866-024-03653-2 39592959 PMC11590571

[13] Husna A Rahman MM Badruzzaman AT Sikder MH Islam MR Rahman MT . Extended-spectrum β-lactamases (ESBL): challenges and opportunities. Biomedicines. (2023) 11:2937. doi: 10.3390/biomedicines11112937 38001938 PMC10669213

[14] Khalifa HO Shikoray L Mohamed MY Habib I Matsumoto T. Veterinary drug residues in the food chain as an emerging public health threat: sources, analytical methods, health impacts, and preventive measures. Foods. (2024) 13:1629. doi: 10.3390/foods13111629 38890858 PMC11172309

[15] Wang J Stephan R Karczmarczyk M Yan Q Hächler H. Molecular characterization of *bla*ESBL–harboring conjugative plasmids identified in multi-drug-resistant *Escherichia coli* isolated from food-producing animals and healthy humans. Front Microbiol. (2013) 4:188. doi: 10.3389/fmicb.2013.00188 23874325 PMC3708134

[16] Habib I Mohamed MY Lakshmi GB Al Marzooqi HM Afifi HS Shehata MG . Quantitative assessment and genomic profiling of *Campylobacter* dynamics in poultry processing: a case study in the United Arab Emirates integrated abattoir system. Front Microbiol. (2024) 15:1439424. doi: 10.3389/fmicb.2024.1439424 39296292 PMC11408311

[17] Clinical and Laboratory Standards Institute (CLSI). Performance standards for antimicrobial susceptibility testing. Wayne, PA: CLSI Supplement M100-S30 (2020).

[18] Fang H Lundberg C Olsson-Liljequist B Hedin G Lindback E Rosenberg Å . Molecular epidemiological analysis of *Escherichia coli* isolates producing extended-spectrum β-lactamases for identification of nosocomial outbreaks in Stockholm, Sweden. J Clin Microbiol. (2004) 42:5917–20. doi: 10.1128/JCM.42.12.5917-5920.2004 15583340 PMC535291

[19] Boyd DA Tyler S Christianson S McGeer A Muller MP Willey BM . Complete nucleotide sequence of a 92-kilobase plasmid harboring the CTX-M-15 extended-spectrum beta-lactamase involved in an outbreak in long-term-care facilities in Toronto, Canada. Antimicrob Agents Chemother. (2004) 48:3758–64. doi: 10.1128/AAC.48.10.3758-3764.2004 15388431 PMC521865

[20] Monstein Ö Nilsson M Nilsson M Dornbusch K Nilsson L. Multiplex PCR amplification assay for the detection of *bla*_SHV_, *bla*_TEM_, and *bla*_CTX−M_ genes in *Enterobacteriaceae*. APMIS. (2007) 115:1400–8. doi: 10.1111/j.1600-0463.2007.00722.x 18184411

[21] Mostatabi N Farshad S Ranjbar R. Molecular evaluations of extended-spectrum β-lactamase-producing strains of *Serratia* isolated from blood samples of patients in Namazi Hospital, Shiraz, Southern Iran. Iran J Microbiol. (2013) 5:328. 25848500 PMC4385156

[22] Bankevich A Nurk S Antipov D Gurevich AA Dvorkin M Kulikov AS . SPAdes: A new genome assembly algorithm and its applications to single-cell sequencing. J Comput Biol. (2012) 19:455–77. doi: 10.1089/cmb.2012.0021 22506599 PMC3342519

[23] Zhou Z Alikhan N-F Mohamed K Fan Y Agama Study G Achtman M . The Enterobase user's guide, with case studies on *Salmonella* transmissions, *Yersinia Pestis* Phylogeny, and Escherichia core genomic diversity. Genome Res. (2020) 30:138–52. doi: 10.1101/gr.251678.119 31809257 PMC6961584

[24] Robertson J Nash JHE. MOB-suite: software tools for clustering, reconstruction and typing of plasmids from draft assemblies. Microb Genom. (2018) 4:e000206. doi: 10.1099/mgen.0.000206 30052170 PMC6159552

[25] Seemann T. Prokka: rapid prokaryotic genome annotation. Bioinformatics. (2014) 30:2068–69. doi: 10.1093/bioinformatics/btu153 24642063

[26] Badr H Reda RM Hagag NM Kamel E Elnomrosy SM Mansour AI . Multidrug-resistant and genetic characterization of extended-spectrum beta-lactamase-producing *E. coli* recovered from chickens and humans in Egypt. Animals. (2022) 12:346. doi: 10.3390/ani12030346 35158668 PMC8833359

[27] Aliyu AB Jalila A Saleha AA. ESBL producing *E.* coli in chickens and poultry farms environment in Selangor, Malaysia: a cross-sectional study on their occurrence and associated risk factors with environment and public health importance. Zoonoses Public Health. (2024) 71:962–71. doi: 10.1111/zph.13179 39289890

[28] Senthamilselvan B Rameshkumar MR Madani ZS Dhanasezhian A Kamalakar S Sivakumar S . Molecular analysis of ESBL- and AmpC-producing *Enterobacteriaceae* in fecal samples from broiler and backyard chickens. J King Saud Univ-Sci. (2024) 36:103191. doi: 10.1016/j.jksus.2024.103191

[29] Eltai NO Abdfarag EA Al-Romaihi H Wehedy E Mahmoud MH Alawad OK . Antibiotic resistance profile of commensal *Escherichia coli* isolated from broiler chickens in Qatar. J Food Prot. (2018) 81:302–7. doi: 10.4315/0362-028X.JFP-17-191 29369690

[30] Caneschi A Bardhi A Barbarossa A Zaghini A. The use of antibiotics and antimicrobial resistance in veterinary medicine, a complex phenomenon: a narrative review. Antibiotics. (2023) 12:487. doi: 10.3390/antibiotics12030487 36978354 PMC10044628

[31] Overdevest I Willemsen I Rijnsburger M Eustace A Xu L Hawkey P . Extended-spectrum β-lactamase genes of *Escherichia coli* in chicken meat and humans, The Netherlands. Emerg Infect Dis. (2011) 17:1216. doi: 10.3201/eid1707.110209 21762575 PMC3381403

[32] Menck-Costa MF Baptista AAS Gazal LES Justino L Sanches MS de Souza M . High-frequency detection of *fosA3* and *bla*_CTX−M−55_ genes in *Escherichia coli* from longitudinal monitoring in broiler chicken farms. Front Microbiol. (2022) 13:846116. doi: 10.3389/fmicb.2022.846116 35663865 PMC9158547

[33] Park H Kim J Ryu S. Predominance of *bla*_CTX−M−65_ and *bla*_CTX−M−55_ in extended-spectrum β-lactamase-producing *Escherichia coli* from raw retail chicken in South Korea. J Glob Antimicrob Resist. (2019) 17:216–20. doi: 10.1016/j.jgar.2019.01.005 30658198

[34] Norizuki C Wachino JI Suzuki M Kawamura K Nagano N Kimura K . Specific *bla*_CTX−M−8_/IncI1 plasmid transfer among genetically diverse *Escherichia coli* isolates between humans and chickens. Antimicrob Agents Chemother. (2017) 61:10–128. doi: 10.1128/AAC.00663-17 PMC5444161 28396551

[35] Zou M Ma PP Liu WS Liang X Li XY Li YZ . Prevalence and antibiotic resistance characteristics of extraintestinal pathogenic *Escherichia coli* among healthy chickens from farms and live poultry markets in China. Animals. (2021) 11:1112. doi: 10.3390/ani11041112 33924454 PMC8070349

[36] Hedman HD Vasco KA. A review of antimicrobial resistance in poultry farming within low-resource settings. Animals. (2020) 10:1264. doi: 10.3390/ani10081264 32722312 PMC7460429

[37] Yang H Chen S White DG Zhao S McDermott P Walker R . Characterization of multiple-antimicrobial-resistant *Escherichia coli* isolates from diseased chickens and swine in China. J Clin Microbiol. (2004) 42:3483–9. doi: 10.1128/JCM.42.8.3483-3489.2004 15297487 PMC497637

[38] He Y Liang B Mai J Lan F Xiong Z Liu X . Distribution of disinfectant-resistant genes in *mcr-1*-carrying *Escherichia coli* isolated from children in southern China. Microb Pathog. (2025) 198:107114. doi: 10.1016/j.micpath.2024.107114 39551109

[39] Carattoli A. Plasmids and the spread of resistance. Int J Med Microbiol. (2013) 303:298–304. doi: 10.1016/j.ijmm.2013.02.001 23499304

[40] Pitout JD. The significance of epidemic plasmids in the success of multidrug-resistant drug pandemic extraintestinal pathogenic *Escherichia coli*. Infect Dis Ther. (2023) 12:1029–41. doi: 10.1007/s40121-023-00791-4 36947392 PMC10147871

[41] Johnson TJ Logue CM Johnson JR Kuskowski MA Sherwood JS Barnes HJ . Associations between multidrug resistance, plasmid content, and virulence potential among extraintestinal pathogenic and commensal *Escherichia coli* from humans and poultry. Foodborne Pathog Dis. (2012) 9:37–46. doi: 10.1089/fpd.2011.0961 21988401 PMC3250628

[42] Fischbach MA Lin H Zhou L Yu Y Abergel RJ Liu DR . The pathogen-associated *iroA* gene cluster mediates bacterial evasion of lipocalin 2. Proc Natl Acad Sci USA. (2006) 103:16502–7. doi: 10.1073/pnas.0604636103 17060628 PMC1637611

[43] Kathayat D Lokesh D Ranjit S Rajashekara G. Avian pathogenic *Escherichia coli* (APEC): an overview of virulence and pathogenesis factors, zoonotic potential, and control strategies. Pathogens. (2021) 10:467. doi: 10.3390/pathogens10040467 33921518 PMC8069529

[44] Coburn B Sekirov I. Type III secretion systems and disease. Clin Microbiol Rev. (2007) 20:535–49. doi: 10.1128/CMR.00013-07 17934073 PMC2176049

[45] Nguyen LT Thuan NK Tam NT Huyen Trang CT Khanh NP Bich TN . Prevalence and genetic relationship of predominant *Escherichia coli* serotypes isolated from poultry, wild animals, and environment in the Mekong Delta, Vietnam. Vet Med Int. (2021) 2021:6504648. doi: 10.1155/2021/6504648 34804471 PMC8601835

[46] Umpiérrez A Bado I Oliver M Acquistapace S Etcheverría A Padola NL . Zoonotic potential and antibiotic resistance of *Escherichia coli* in neonatal calves in Uruguay. Microbes Environ. (2017) 32:275–82. doi: 10.1264/jsme2.ME17046 28904264 PMC5606698

[47] Salim A Babu P Mohan K Moorthy M Raj D Kallampillil Thirumeni S . Draft genome sequence of an *Escherichia coli* sequence type 155 strain isolated from sewage in Kerala, India. Microbiol Resour Announc. (2019) 8:10–128. doi: 10.1128/MRA.01707-18 31270207 PMC6606921

[48] Ramos S Silva V Dapkevicius MD Caniça M Tejedor-Junco MT Igrejas G . *Escherichia coli* as commensal and pathogenic bacteria among food-producing animals: health implications of extended-spectrum β-lactamase (ESBL) production. Animals. (2020) 10:2239. doi: 10.3390/ani10122239 33260303 PMC7761174

