# Supplementary material for: Prevalence and genetic basis of extended-spectrum β-lactamase-producing Escherichia coli carriage in broiler farms in the United Arab Emirates
Source: Front Vet Sci. 2025 Dec 8;12:1714381. doi: 10.3389/fvets.2025.1714381 (PMC12723266; doi:10.3389/fvets.2025.1714381)
Supplement: Supplementary file 1 [file Data_Sheet_1.docx]

Supplementary Material

**Supplementary Table S1**: Oligonucleotides used in this study

| Primer name | Primer sequence (5′ to 3′) | Amplicon size | Target gene |  | Reference |
| --- | --- | --- | --- | --- | --- |
| blaSHV-F | CTTTATCGGCCCTCACTCAA | 237 | *bla*_SHV_ | B-lactams genes | Fang et al., 2004 |
| blaSHV-R | AGGTGCTCATCATGGGAAAG |  |  |  |  |
| blaTEM-F | CGCCGCATACACTATTCTCAGAATGA | 455 | *bla*_TEM_ |  | Monstein et al., 2007 |
| blaTEM-R | ACGCTCACCGGCTCCAGATTTAT |  |  |  |  |
| blaCTX-M-F | ATGTGCAGYACCAGTAARGTKATGGC | 593 | *bla*_CTX_ |  | Boyd et al., 2004 |
| blaCTX-M-R | TGGGTRAARTARGTSACCAGAAYCAGCGG |  |  |  |  |
| blaOXA-F | ACACAATACATATCAACTTCGC | 813 | *bla*_OXA_ |  | Boyd et al., 2013 |
| blaOXA-R | AGT GTG TTT AGA ATG GTG ATC |  |  |  |  |
| CTXM7 | GCGTGATACCACTTCACCTC | 260 | *bla*_CTX-1 group_ | Specific for *bla*_CTX_ | Khalifa et al., 2021a |
| CTXM8 | TGAAGTAAGTGACCAGAATC |  |  |  |  |
| CTXM17 | TGATACCACCACGCCGCT C | 341 | *bla*_CTX-2 group_ |  |  |
| CTXM18 | TATTGCATCAGAAACCGTGGG |  |  |  |  |
| CTXM11 | ATCAAGCCTGCCGATCTGGTTA | 293 | *bla*_CTX-9 group_ |  |  |
| CTXM12 | GTAAGCTGACGCAACGTCTGC |  |  |  |  |

**Supplementary Table S2:** List of the isolates used for the phylogenetic analysis

| Strain | Name | Data Source(Accession No. | Source Niche | Source Type | Source Details | Collection Year | Country | Bio Project ID | Sample ID | MLST |
| --- | --- | --- | --- | --- | --- | --- | --- | --- | --- | --- |
| ESC_YA5755AA | FD5-2 | ERR7528890 |  |  | Human |  | United Arab Emirates | PRJEB49171 | SAMEA11421029 | ST1011 |
| ESC_YA5756AA | FD4-4 | ERR7528889 |  |  | Human |  | United Arab Emirates | PRJEB49171 | SAMEA11421027 | ST1630 |
| ESC_YA5758AA | FD2-7 | ERR7528886 |  |  | Human |  | United Arab Emirates | PRJEB49171 | SAMEA11421025 | ST165 |
| ESC_YA5759AA | FD10-8 | ERR7528885 |  |  | Human |  | United Arab Emirates | PRJEB49171 | SAMEA11421024 | ST354 |
| ESC_YA5760AA | FD10-2 | ERR7528884 |  |  | Human |  | United Arab Emirates | PRJEB49171 | SAMEA11421023 | ST354 |
| ESC_YA5761AA | FC9-8 | ERR7528882 |  |  | Human |  | United Arab Emirates | PRJEB49171 | SAMEA11421022 | ST1485 |
| ESC_YA5762AA | FC9-1 | ERR7528881 |  |  | Human |  | United Arab Emirates | PRJEB49171 | SAMEA11421021 | ST1585 |
| ESC_YA5763AA | FC6-10-1 | ERR7528880 |  |  | Human |  | United Arab Emirates | PRJEB49171 | SAMEA11421020 | ST1585 |
| ESC_YA5764AA | FC5-3 | ERR7528878 |  |  | Human |  | United Arab Emirates | PRJEB49171 | SAMEA11421019 | ST1485 |
| ESC_YA5765AA | FC5-2 | ERR7528877 |  |  | Human |  | United Arab Emirates | PRJEB49171 | SAMEA11421018 | ST1485 |
| ESC_YA5766AA | FC5-1 | ERR7528876 |  |  | Human |  | United Arab Emirates | PRJEB49171 | SAMEA11421017 | ST1585 |
| ESC_YA5767AA | FC4-8 | ERR7528875 |  |  | Human |  | United Arab Emirates | PRJEB49171 | SAMEA11421016 | ST93 |
| ESC_YA5768AA | FC3-1 | ERR7528874 |  |  | Human |  | United Arab Emirates | PRJEB49171 | SAMEA11421015 | ST1485 |
| ESC_YA5770AA | FC10-1 | ERR7528872 |  |  | Human |  | United Arab Emirates | PRJEB49171 | SAMEA11421013 | ST162 |
| ESC_YA5771AA | FC1-3 | ERR7528871 |  |  | Human |  | United Arab Emirates | PRJEB49171 | SAMEA11421012 | ST354 |
| ESC_YA5772AA | FC1-2-1 | ERR7528870 |  |  | Human |  | United Arab Emirates | PRJEB49171 | SAMEA11421011 | ST1585 |
| ESC_YA5773AA | FB7-5 | ERR7528868 |  |  | Human |  | United Arab Emirates | PRJEB49171 | SAMEA11421010 | ST1140 |
| ESC_YA5774AA | FB7-1 | ERR7528867 |  |  | Human |  | United Arab Emirates | PRJEB49171 | SAMEA11421009 | ST1196 |
| ESC_YA5775AA | FB3-1 | ERR7528866 |  |  | Human |  | United Arab Emirates | PRJEB49171 | SAMEA11421008 | ST1140 |
| ESC_YA5776AA | FB2-1 | ERR7528865 |  |  | Human |  | United Arab Emirates | PRJEB49171 | SAMEA11421007 | ST69 |
| ESC_YA5777AA | FB1-1 | ERR7528863 |  |  | Human |  | United Arab Emirates | PRJEB49171 | SAMEA11421006 | ST1196 |
| ESC_YA5778AA | FA7-9 | ERR7528862 |  |  | Human |  | United Arab Emirates | PRJEB49171 | SAMEA11421005 | ST1290 |
| ESC_YA5779AA | FA6-1 | ERR7528861 |  |  | Human |  | United Arab Emirates | PRJEB49171 | SAMEA11421004 | ST12220 |
| ESC_YA5780AA | FA5-1 | ERR7528860 |  |  | Human |  | United Arab Emirates | PRJEB49171 | SAMEA11421003 | ST12220 |
| ESC_YA5781AA | FD9-7-1 | ERR7460975 |  |  | Human |  | United Arab Emirates | PRJEB49171 | SAMEA11358404 | ST101 |
| ESC_YA5782AA | FD8-8 | ERR7460974 |  |  | Human |  | United Arab Emirates | PRJEB49171 | SAMEA11358403 | ST101 |
| ESC_YA5783AA | FD7-1-1 | ERR7460972 |  |  | Human |  | United Arab Emirates | PRJEB49171 | SAMEA11358402 | ST101 |
| ESC_YA5784AA | FD1-4 | ERR7460948 |  |  | Human |  | United Arab Emirates | PRJEB49171 | SAMEA11358378 | ST101 |
| ESC_YA5785AA | FC2-9-1 | ERR7460947 |  |  | Human |  | United Arab Emirates | PRJEB49171 | SAMEA11358377 | ST533 |
| ESC_YA5786AA | FA4-9 | ERR7460946 |  |  | Human |  | United Arab Emirates | PRJEB49171 | SAMEA11358376 | ST48 |
| ESC_YA5787AA | FA1-1 | ERR7460945 |  |  | Human |  | United Arab Emirates | PRJEB49171 | SAMEA11358375 | ST48 |
| ESC_YA5789AA | FB10-1 | ERR7460208 |  |  | Human |  | United Arab Emirates | PRJEB49171 | SAMEA11356967 | ST101 |
| ESC_AB5780AA | microbial | SRR20708239 | Environment | ND/Others | environemental | 2020 | United Arab Emirates | PRJNA862939 | SAMN30002575 | ST189 |
| ESC_AB5781AA | microbial | SRR20708240 | Environment | ND/Others | environemental | 2020 | United Arab Emirates | PRJNA862939 | SAMN30002574 | ST359 |
| ESC_AB5782AA | microbial | SRR20708241 | Environment | ND/Others | environemental | 2020 | United Arab Emirates | PRJNA862939 | SAMN30002573 | ST359 |
| ESC_AB5783AA | microbial | SRR20708242 | Environment | ND/Others | environemental | 2020 | United Arab Emirates | PRJNA862939 | SAMN30002572 | ST10 |
| ESC_AB5784AA | microbial | SRR20708243 | Environment | ND/Others | environemental | 2020 | United Arab Emirates | PRJNA862939 | SAMN30002571 | ST189 |
| ESC_AB5785AA | microbial | SRR20708244 | Environment | ND/Others | environemental | 2020 | United Arab Emirates | PRJNA862939 | SAMN30002570 | ST1158 |
| ESC_AB5786AA | microbial | SRR20708245 | Environment | ND/Others | environemental | 2020 | United Arab Emirates | PRJNA862939 | SAMN30002569 | ST10 |
| ESC_AB5787AA | microbial | SRR20708246 | Environment | ND/Others | environemental | 2021 | United Arab Emirates | PRJNA862939 | SAMN30002592 | ST1011 |
| ESC_AB5788AA | microbial | SRR20708247 | Environment | ND/Others | environemental | 2021 | United Arab Emirates | PRJNA862939 | SAMN30002591 | ST602 |
| ESC_AB5789AA | microbial | SRR20708248 | Environment | ND/Others | environemental | 2021 | United Arab Emirates | PRJNA862939 | SAMN30002590 | ST1011 |
| ESC_AB5790AA | microbial | SRR20708249 | Environment | ND/Others | environemental | 2021 | United Arab Emirates | PRJNA862939 | SAMN30002589 | ST1011 |
| ESC_AB5791AA | microbial | SRR20708250 | Environment | ND/Others | environemental | 2021 | United Arab Emirates | PRJNA862939 | SAMN30002588 | ST10 |
| ESC_AB5792AA | microbial | SRR20708251 | Environment | ND/Others | environemental | 2021 | United Arab Emirates | PRJNA862939 | SAMN30002587 | ST13474 |
| ESC_AB5793AA | microbial | SRR20708252 | Environment | ND/Others | environemental | 2021 | United Arab Emirates | PRJNA862939 | SAMN30002586 | ST1011 |
| ESC_AB5794AA | microbial | SRR20708253 | Environment | ND/Others | environemental | 2020 | United Arab Emirates | PRJNA862939 | SAMN30002568 | ST359 |
| ESC_AB5795AA | microbial | SRR20708254 | Environment | ND/Others | environemental | 2020 | United Arab Emirates | PRJNA862939 | SAMN30002585 | ST38 |
| ESC_AB5796AA | microbial | SRR20708255 | Environment | ND/Others | environemental | 2020 | United Arab Emirates | PRJNA862939 | SAMN30002584 | ST354 |
| ESC_AB5797AA | microbial | SRR20708256 | Environment | ND/Others | environemental | 2020 | United Arab Emirates | PRJNA862939 | SAMN30002583 | ST1140 |
| ESC_AB5798AA | microbial | SRR20708257 | Environment | ND/Others | environemental | 2020 | United Arab Emirates | PRJNA862939 | SAMN30002582 | ST93 |
| ESC_AB5799AA | microbial | SRR20708258 | Environment | ND/Others | environemental | 2020 | United Arab Emirates | PRJNA862939 | SAMN30002581 | ST1011 |
| ESC_AB5800AA | microbial | SRR20708259 | Environment | ND/Others | environemental | 2020 | United Arab Emirates | PRJNA862939 | SAMN30002580 | ST1290 |
| ESC_AB5801AA | microbial | SRR20708260 | Environment | ND/Others | environemental | 2020 | United Arab Emirates | PRJNA862939 | SAMN30002579 | ST212 |
| ESC_AB5802AA | microbial | SRR20708261 | Environment | ND/Others | environemental | 2020 | United Arab Emirates | PRJNA862939 | SAMN30002578 | ST189 |
| ESC_AB5803AA | microbial | SRR20708262 | Environment | ND/Others | environemental | 2020 | United Arab Emirates | PRJNA862939 | SAMN30002577 | ST752 |
| ESC_AB5804AA | microbial | SRR20708263 | Environment | ND/Others | environemental | 2020 | United Arab Emirates | PRJNA862939 | SAMN30002576 | ST359 |
| ESC_AB5805AA | microbial | SRR20708264 | Environment | ND/Others | environemental | 2020 | United Arab Emirates | PRJNA862939 | SAMN30002567 | ST189 |
| ESC_AB5806AA | microbial | SRR20708265 | Environment | ND/Others | environemental | 2020 | United Arab Emirates | PRJNA862939 | SAMN30002566 | ST359 |
| ESC_DB8009AA | Microbial | SRR23622528 | Companion Animal | Dog | rectal swab | 2005 | United Arab Emirates | PRJNA938811 | SAMN33422834 | ST1011 |
| ESC_DB8010AA | Microbial | SRR23622529 | Companion Animal | Cat | rectal swab | 2005 | United Arab Emirates | PRJNA938811 | SAMN33422833 | ST1011 |
| ESC_FB3294AA | ECV-10 | SRR24631097 | Environment | Plant | Arugula | 2023 | United Arab Emirates | PRJNA973558 | SAMN35129925 | ST7588 |
| ESC_FB3295AA | ECV-9 | SRR24631098 | Environment | Plant | Arugula | 2023 | United Arab Emirates | PRJNA973558 | SAMN35129924 | ST7588 |
| ESC_FB3296AA | ECV-8 | SRR24631099 | Environment | Plant | Rocket | 2023 | United Arab Emirates | PRJNA973558 | SAMN35129923 | ST58 |
| ESC_FB3297AA | ECV-7 | SRR24631100 | Food | Plant | Spinach | 2023 | United Arab Emirates | PRJNA973558 | SAMN35129922 | ST10 |
| ESC_FB3298AA | ECV-6 | SRR24631101 | Environment | Plant | Parsley | 2023 | United Arab Emirates | PRJNA973558 | SAMN35129921 | ST2161 |
| ESC_FB3299AA | ECV-5 | SRR24631102 | Environment | Plant | Corriander | 2023 | United Arab Emirates | PRJNA973558 | SAMN35129920 | ST1727 |
| ESC_FB3300AA | ECV-4 | SRR24631103 | Environment | Plant | Parsley | 2023 | United Arab Emirates | PRJNA973558 | SAMN35129919 | ST1294 |
| ESC_FB3301AA | ECV-3 | SRR24631104 | Environment | Plant | Arugula | 2023 | United Arab Emirates | PRJNA973558 | SAMN35129918 | ST155 |
| ESC_FB3302AA | ECV-20 | SRR24631105 | Environment | Plant | Parsley | 2023 | United Arab Emirates | PRJNA973558 | SAMN35129933 | ST224 |
| ESC_FB3303AA | ECV-19 | SRR24631106 | Environment | Plant | Arugula | 2023 | United Arab Emirates | PRJNA973558 | SAMN35129932 | ST328 |
| ESC_FB3304AA | ECV-18 | SRR24631107 | Environment | Plant | Arugula | 2023 | United Arab Emirates | PRJNA973558 | SAMN35129931 | ST7588 |
| ESC_FB3305AA | ECV-16 | SRR24631108 | Environment | Plant | Corriander | 2023 | United Arab Emirates | PRJNA973558 | SAMN35129930 | ST2206 |
| ESC_FB3306AA | ECV-14 | SRR24631109 | Environment | Plant | Parsley | 2023 | United Arab Emirates | PRJNA973558 | SAMN35129929 | ST206 |
| ESC_FB3307AA | ECV-13 | SRR24631110 | Environment | Plant | Lettuce | 2023 | United Arab Emirates | PRJNA973558 | SAMN35129928 | ST58 |
| ESC_FB3308AA | ECV-12 | SRR24631111 | Environment | Plant | Parsley | 2023 | United Arab Emirates | PRJNA973558 | SAMN35129927 | ST206 |
| ESC_FB3309AA | ECV-11 | SRR24631112 | Environment | Plant | Parsley | 2023 | United Arab Emirates | PRJNA973558 | SAMN35129926 | ST1642 |
| ESC_FB3310AA | ECV-2 | SRR24631113 | Environment | Plant | Arugula | 2023 | United Arab Emirates | PRJNA973558 | SAMN35129917 | ST1727 |
| ESC_FB3311AA | ECV-1 | SRR24631114 | Environment | Plant | Arugula | 2023 | United Arab Emirates | PRJNA973558 | SAMN35129916 | ST58 |
| ESC_QB0685AA | ECF31 | SRR32234830 | Poultry | Poultry | Broiler carcass | 2023 | United Arab Emirates | PRJNA1219370 | SAMN46555831 | ST2562 |
| ESC_QB0686AA | ECF30 | SRR32234831 | Poultry | Poultry | Broiler carcass | 2023 | United Arab Emirates | PRJNA1219370 | SAMN46555830 | ST602 |
| ESC_QB0687AA | ECF29 | SRR32234832 | Poultry | Poultry | Broiler carcass | 2023 | United Arab Emirates | PRJNA1219370 | SAMN46555829 | ST101 |
| ESC_QB0688AA | ECF27 | SRR32234833 | Poultry | Poultry | Broiler carcass | 2023 | United Arab Emirates | PRJNA1219370 | SAMN46555828 | ST191 |
| ESC_QB0689AA | ECF26 | SRR32234834 | Poultry | Poultry | Broiler carcass | 2023 | United Arab Emirates | PRJNA1219370 | SAMN46555827 | ST4753 |
| ESC_QB0690AA | ECF25 | SRR32234835 | Poultry | Poultry | Broiler carcass | 2023 | United Arab Emirates | PRJNA1219370 | SAMN46555826 | ST58 |
| ESC_QB0691AA | ECF55 | SRR32234836 | Poultry | Poultry | Broiler carcass | 2023 | United Arab Emirates | PRJNA1219370 | SAMN46555854 | ST10 |
| ESC_QB0692AA | ECF54 | SRR32234837 | Poultry | Poultry | Broiler carcass | 2023 | United Arab Emirates | PRJNA1219370 | SAMN46555853 | ST4566 |
| ESC_QB0693AA | ECF53 | SRR32234838 | Poultry | Poultry | Broiler carcass | 2023 | United Arab Emirates | PRJNA1219370 | SAMN46555852 | ST38 |
| ESC_QB0694AA | ECF24 | SRR32234839 | Poultry | Poultry | Broiler carcass | 2023 | United Arab Emirates | PRJNA1219370 | SAMN46555825 | ST115 |
| ESC_QB0695AA | ECF52 | SRR32234840 | Poultry | Poultry | Broiler carcass | 2023 | United Arab Emirates | PRJNA1219370 | SAMN46555851 | ST1564 |
| ESC_QB0696AA | ECF51 | SRR32234841 | Poultry | Poultry | Broiler carcass | 2023 | United Arab Emirates | PRJNA1219370 | SAMN46555850 | ST69 |
| ESC_QB0697AA | ECF50 | SRR32234842 | Poultry | Poultry | Broiler carcass | 2023 | United Arab Emirates | PRJNA1219370 | SAMN46555849 | ST770 |
| ESC_QB0698AA | ECF49 | SRR32234843 | Poultry | Poultry | Broiler carcass | 2023 | United Arab Emirates | PRJNA1219370 | SAMN46555848 | ST1564 |
| ESC_QB0699AA | ECF48 | SRR32234844 | Poultry | Poultry | Broiler carcass | 2023 | United Arab Emirates | PRJNA1219370 | SAMN46555847 | ST115 |
| ESC_QB0700AA | ECF47 | SRR32234845 | Poultry | Poultry | Broiler carcass | 2023 | United Arab Emirates | PRJNA1219370 | SAMN46555846 | ST101 |
| ESC_QB0701AA | ECF46 | SRR32234846 | Poultry | Poultry | Broiler carcass | 2023 | United Arab Emirates | PRJNA1219370 | SAMN46555845 | ST1564 |
| ESC_QB0702AA | ECF45 | SRR32234847 | Poultry | Poultry | Broiler carcass | 2023 | United Arab Emirates | PRJNA1219370 | SAMN46555844 | ST115 |
| ESC_QB0703AA | ECF44 | SRR32234848 | Poultry | Poultry | Broiler carcass | 2023 | United Arab Emirates | PRJNA1219370 | SAMN46555843 | ST4243 |
| ESC_QB0704AA | ECF43 | SRR32234849 | Poultry | Poultry | Broiler carcass | 2023 | United Arab Emirates | PRJNA1219370 | SAMN46555842 | ST4110 |
| ESC_QB0705AA | ECF23 | SRR32234850 | Poultry | Poultry | Broiler carcass | 2023 | United Arab Emirates | PRJNA1219370 | SAMN46555824 | ST1266 |
| ESC_QB0706AA | ECF42 | SRR32234851 | Poultry | Poultry | Broiler carcass | 2023 | United Arab Emirates | PRJNA1219370 | SAMN46555841 | ST155 |
| ESC_QB0707AA | ECF41 | SRR32234852 | Poultry | Poultry | Broiler carcass | 2023 | United Arab Emirates | PRJNA1219370 | SAMN46555840 | ST1101 |
| ESC_QB0708AA | ECF40 | SRR32234853 | Poultry | Poultry | Broiler carcass | 2023 | United Arab Emirates | PRJNA1219370 | SAMN46555839 | ST1720 |
| ESC_QB0709AA | ECF39 | SRR32234854 | Poultry | Poultry | Broiler carcass | 2023 | United Arab Emirates | PRJNA1219370 | SAMN46555838 | ST48 |
| ESC_QB0710AA | ECF38 | SRR32234855 | Poultry | Poultry | Broiler carcass | 2023 | United Arab Emirates | PRJNA1219370 | SAMN46555837 | ST69 |
| ESC_QB0711AA | ECF37 | SRR32234856 | Poultry | Poultry | Broiler carcass | 2023 | United Arab Emirates | PRJNA1219370 | SAMN46555836 | ST155 |
| ESC_QB0712AA | ECF36 | SRR32234857 | Poultry | Poultry | Broiler carcass | 2023 | United Arab Emirates | PRJNA1219370 | SAMN46555835 | ST1011 |
| ESC_QB0713AA | ECF35 | SRR32234858 | Poultry | Poultry | Broiler carcass | 2023 | United Arab Emirates | PRJNA1219370 | SAMN46555834 | ST1564 |
| ESC_QB0714AA | ECF34 | SRR32234859 | Poultry | Poultry | Broiler carcass | 2023 | United Arab Emirates | PRJNA1219370 | SAMN46555833 | ST4753 |
| ESC_QB0715AA | ECF32 | SRR32234860 | Poultry | Poultry | Broiler carcass | 2023 | United Arab Emirates | PRJNA1219370 | SAMN46555832 | ST58 |
| ESC_QB0716AA | ECF22 | SRR32234861 | Poultry | Poultry | Broiler carcass | 2023 | United Arab Emirates | PRJNA1219370 | SAMN46555823 | ST8132 |
| ESC_QB0717AA | ECF21 | SRR32234862 | Poultry | Poultry | Broiler carcass | 2023 | United Arab Emirates | PRJNA1219370 | SAMN46555822 | ST1158 |
| ESC_QB1409AA | S314C2 | SRR32268887 | Poultry | Poultry | Chicken cecal content | 2024 | United Arab Emirates | PRJNA1220707 | SAMN46723440 | ST1011 |
| ESC_QB1410AA | S314C1 | SRR32268888 | Poultry | Poultry | Chicken cecal content | 2024 | United Arab Emirates | PRJNA1220707 | SAMN46723439 | ST155 |
| ESC_QB1411AA | S316C | SRR32268889 | Poultry | Poultry | Chicken ecal dropping | 2024 | United Arab Emirates | PRJNA1220707 | SAMN46723438 | ST694 |
| ESC_QB1412AA | S279C | SRR32268890 | Poultry | Poultry | Chicken ecal dropping | 2024 | United Arab Emirates | PRJNA1220707 | SAMN46723437 | ST10 |
| ESC_QB1413AA | S259C | SRR32268891 | Poultry | Poultry | Chicken ecal dropping | 2023 | United Arab Emirates | PRJNA1220707 | SAMN46723436 | ST694 |
| ESC_QB1414AA | S203C | SRR32268892 | Poultry | Poultry | Chicken ecal dropping | 2023 | United Arab Emirates | PRJNA1220707 | SAMN46723435 | ST694 |
| ESC_QB1415AA | S202C | SRR32268893 | Poultry | Poultry | Chicken ecal dropping | 2023 | United Arab Emirates | PRJNA1220707 | SAMN46723434 | ST694 |
| ESC_QB1416AA | S199C | SRR32268894 | Poultry | Poultry | Chicken cecal content | 2023 | United Arab Emirates | PRJNA1220707 | SAMN46723433 | ST694 |
| ESC_QB1417AA | S377C | SRR32268895 | Poultry | Poultry | Chicken ecal dropping | 2024 | United Arab Emirates | PRJNA1220707 | SAMN46723446 | ST155 |
| ESC_QB1418AA | S367C | SRR32268896 | Poultry | Poultry | Chicken ecal dropping | 2024 | United Arab Emirates | PRJNA1220707 | SAMN46723445 | ST57 |
| ESC_QB1419AA | S358C | SRR32268897 | Poultry | Poultry | Chicken ecal dropping | 2024 | United Arab Emirates | PRJNA1220707 | SAMN46723444 | ST155 |
| ESC_QB1420AA | S357C | SRR32268898 | Poultry | Poultry | Chicken ecal dropping | 2024 | United Arab Emirates | PRJNA1220707 | SAMN46723443 | ST694 |
| ESC_QB1421AA | S336C | SRR32268899 | Poultry | Poultry | Chicken ecal dropping | 2024 | United Arab Emirates | PRJNA1220707 | SAMN46723442 | ST6751 |
| ESC_QB1422AA | S335C2 | SRR32268900 | Poultry | Poultry | Chicken ecal dropping | 2024 | United Arab Emirates | PRJNA1220707 | SAMN46723441 | ST215 |
| ESC_QB1423AA | S136C1 | SRR32268901 | Poultry | Poultry | Chicken cecal content | 2023 | United Arab Emirates | PRJNA1220707 | SAMN46723432 | ST3489 |
| ESC_QB1424AA | S64C2 | SRR32268902 | Poultry | Poultry | Chicken cecal content | 2023 | United Arab Emirates | PRJNA1220707 | SAMN46723431 | ST57 |
| ESC_QB1439AA | S167C1 | SRR32272987 | Poultry | Poultry | Chicken ecal dropping | 2023 | United Arab Emirates | PRJNA1221085 | SAMN46727108 | ST10 |
| ESC_QB1440AA | S166C2 | SRR32272988 | Poultry | Poultry | Chicken ecal dropping | 2023 | United Arab Emirates | PRJNA1221085 | SAMN46727107 | ST354 |
| ESC_QB1441AA | S166C1 | SRR32272989 | Poultry | Poultry | Chicken ecal dropping | 2023 | United Arab Emirates | PRJNA1221085 | SAMN46727106 | ST101 |
| ESC_QB1442AA | S165C | SRR32272990 | Poultry | Poultry | Chicken ecal dropping | 2023 | United Arab Emirates | PRJNA1221085 | SAMN46727105 | ST162 |
| ESC_QB1443AA | S136C2 | SRR32272991 | Poultry | Poultry | Chicken cecal content | 2023 | United Arab Emirates | PRJNA1221085 | SAMN46727104 | ST457 |
| ESC_QB1444AA | S131C | SRR32272992 | Poultry | Poultry | Chicken cecal content | 2023 | United Arab Emirates | PRJNA1221085 | SAMN46727103 | ST117 |
| ESC_QB1445AA | S115C | SRR32272993 | Poultry | Poultry | Chicken cecal content | 2023 | United Arab Emirates | PRJNA1221085 | SAMN46727102 | ST359 |
| ESC_QB1446AA | S99C | SRR32272994 | Poultry | Poultry | Chicken cecal content | 2023 | United Arab Emirates | PRJNA1221085 | SAMN46727101 | ST117 |
| ESC_QB1447AA | S296C | SRR32272995 | Poultry | Poultry | Chicken ecal dropping | 2024 | United Arab Emirates | PRJNA1221085 | SAMN46727113 | ST10 |
| ESC_QB1448AA | S261C1 | SRR32272996 | Poultry | Poultry | Chicken ecal dropping | 2023 | United Arab Emirates | PRJNA1221085 | SAMN46727112 | ST195 |
| ESC_QB1449AA | S200C2 | SRR32272997 | Poultry | Poultry | Chicken ecal dropping | 2023 | United Arab Emirates | PRJNA1221085 | SAMN46727111 | ST162 |
| ESC_QB1450AA | S200C1 | SRR32272998 | Poultry | Poultry | Chicken ecal dropping | 2023 | United Arab Emirates | PRJNA1221085 | SAMN46727110 | ST1771 |
| ESC_QB1451AA | S167C2 | SRR32272999 | Poultry | Poultry | Chicken ecal dropping | 2023 | United Arab Emirates | PRJNA1221085 | SAMN46727109 | ST2172 |
| ESC_QB1452AA | S64C1 | SRR32273000 | Poultry | Poultry | Chicken cecal content | 2023 | United Arab Emirates | PRJNA1221085 | SAMN46727100 | ST46 |
| ESC_QB1453AA | S48C | SRR32273001 | Poultry | Poultry | Chicken cecal content | 2023 | United Arab Emirates | PRJNA1221085 | SAMN46727099 | ST189 |
|  |  |  |  |  |  |  | Reference |  | SAMN02604091 | ST10 |

**Supplementary S3**: Number of antimicrobial resistant genes in *E. coli* tested in this study based on whole genome sequencing of 31 selected isolates

| **Antibiotic group** | **Resistance genes** | **Number** |
| --- | --- | --- |
| **β-lactams** | *bla*_TEM_ | 23 |
|  | *bla*_CTX-M_ | 26 |
|  | *bla*_SHV_ | 1 |
|  | *bla*_CMY_ | 1 |
| **Aminoglycosides** | *aph(3'')-Ib* | 15 |
|  | *aadA2* | 10 |
|  | *aadA24* | 3 |
|  | *aadA22* | 2 |
|  | *aph(6)-Id* | 16 |
|  | *aadA1* | 8 |
|  | *aadA1* | 1 |
|  | *aph(3')-Ia* | 17 |
|  | *aac(3)-Iid* | 7 |
|  | *ant(3'')-Ia* | 8 |
|  | *aac(3)-Iia* | 6 |
|  | *aac(3)-IV* | 1 |
|  | *aph(4)-Ia* | 1 |
|  | *ant(2'')-Ia* | 2 |
|  | aac(3)-Via | 1 |
| **Quinolone** | *qnrS1* | 11 |
|  | *qnrS13* | 1 |
| **Trimethoprim** | *dfrA12* | 2 |
|  | *dfrA14* | 10 |
|  | *dfrA1* | 3 |
|  | *dfrA17* | 1 |
| **Sulfonamides** | *sul1* | 5 |
|  | *sul2* | 14 |
|  | *sul3* | 18 |
| **Tetracycline** | *tet(A)* | 22 |
|  | *tet(M)* | 1 |
| **Phenicol** | *floR* | 11 |
|  | *catA1* | 3 |
|  | *cmlA1* | 12 |
| **Macrolide resistance** | *mph(A)* | 6 |
|  | *erm(B)* | 4 |
| **Rifampicin** | *arr-2* | 1 |
| **Colistin** | *mcr-1.1* | 15 |
| **Fosfomycin** | *fosA4* | 8 |
|  | *fosA3* | 6 |
| **Lincosamides** | *lnu(F)* | 2 |
| **Disinfectant** | *qacE* | 4 |
|  | *sitABCD* | 22 |

**Supplementary S4**: Common identified *E. coli* STs in this study based on whole genome sequencing of 31 selected isolates

| **ST** | **Isolate** | **Isolation date** | **Source** | **Farm** |
| --- | --- | --- | --- | --- |
| **ST694** | S199C | 21/11/2023 | Cecal contents | First poultry farm  Farm E (slaughter house)  House 3 |
|  | S202C | 23/11/2023 | Fecal drop | First poultry farm  Farm F  House 4 |
|  | S203C | 23/11/2023 | Fecal drop | First poultry farm  Farm F  House 1 |
|  | S259C | 19/12/2023 | Fecal drop | First poultry farm  Farm C  House 1 |
|  | S357C | 12/03/2024 | Fecal drop | First poultry farm  Farm B  House 1 |
| **ST10** | S167C1 | 09/11/2023 | Fecal drop | Second poultry farm  Farm 3  House 15 |
|  | S279C | 30/01/2024 | Fecal drop | First poultry farm  Farm C  House 4 |
|  | S296C | 13/02/2024 | Fecal drop | First poultry farm  Farm D  House 7 |
| **ST155** | S314C | 14/02/2024 | Cecal Content | First poultry farm  Farm C  House 4 |
|  | S358C | 12/03/2024 | Fecal drop | First poultry farm  Farm B  House 4 |
|  | S377C | 02/04/2024 | Fecal drop | First poultry farm  Farm E  House 7 |
| **ST117** | S99C | 14/08/2023 | Cecal Content | Second poultry farm  Farm 2  House 10 |
|  | S131C | 28/08/2023 | Cecal Content | Second poultry farm  Farm 5  House 35 |
| **ST162** | S165C | 10/10/2023 | Fecal dropping | Second poultry farm  Farm 5  House 35 |
|  | S200C2 | 21/11/2023 | Fecal dropping | First poultry farm  Farm 5  House 35 |
| **ST195** | S261C1 | 26/12/2023 | Fecal drop | First poultry farm  Farm D  House 7 |
|  | S261C2 |  |  |  |
| **ST2172** | S167C2 | 09/11/2023 | Fecal drop | Second poultry farm  Farm 3  House 15 |
|  | S167C3 |  |  |  |


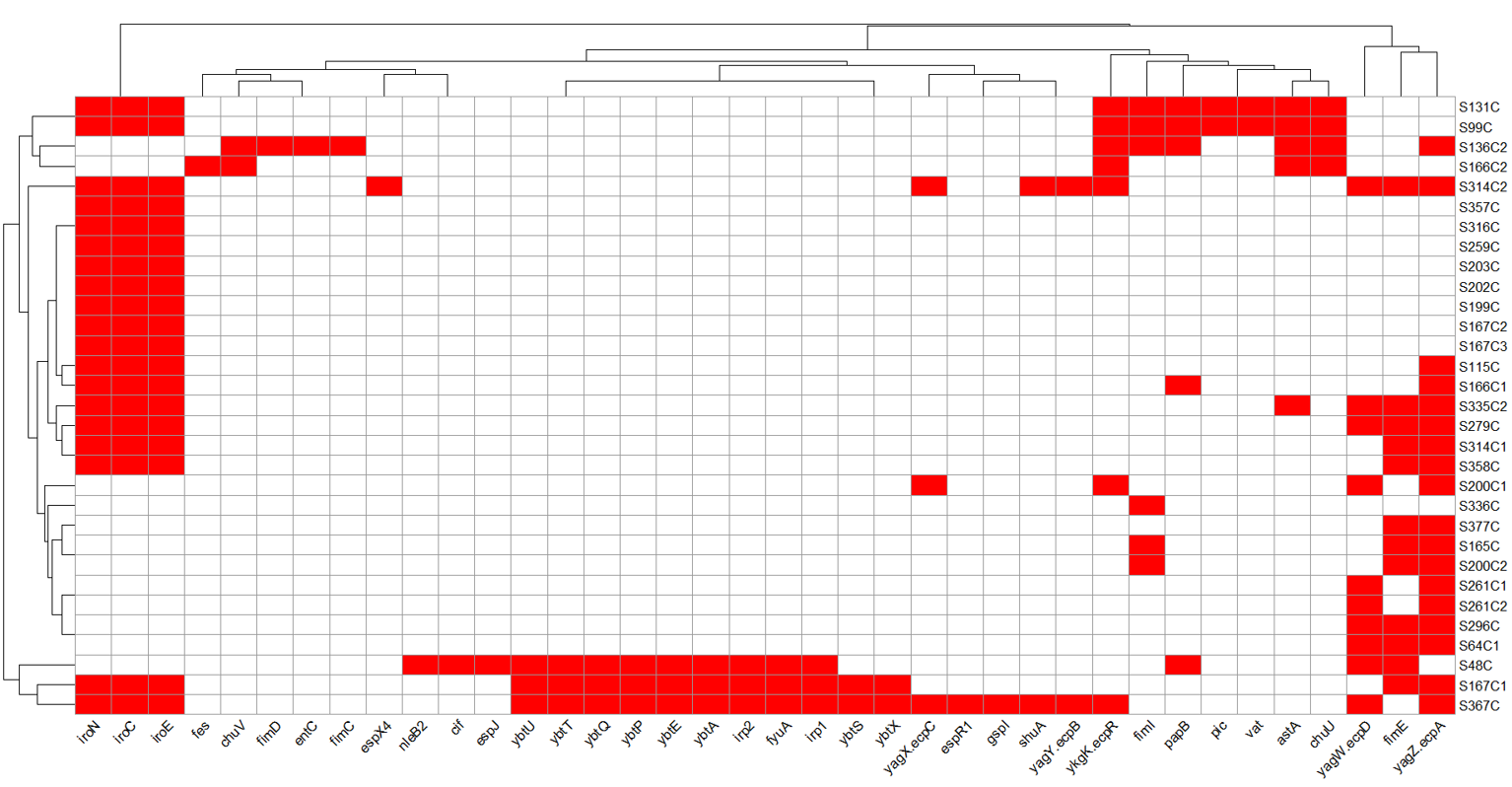
**Supplementary Fig. S1**: Distribution of virulence genes in *E. coli* isolates. Red markings indicate the presence of a virulence genes, while white markings denote its absence. The samples are arranged by isolate ID.

References

Fang, H., Lundberg, C., Olsson-Liljequist, B., et al. 2004. Molecular epidemiological analysis of *Escherichia coli* isolates producing extended-spectrum β-lactamases for identification of nosocomial outbreaks in Stockholm, Sweden. *J Clin Microbiol*, 42(12), 5917-5920. <https://doi.org/10.1128/JCM.42.12.5917-5920.2004>.

Monstein, Ö., Nilsson, M., Nilsson, M., Dornbusch, K., Nilsson, L. 2007. Multiplex PCR amplification assay for the detection of *bla*_SHV_, *bla*_TEM_, and *bla*_CTX‐M_ genes in *Enterobacteriaceae*. *APMIS*, 115, 1400–1408. doi: 10.1111/j.1600-0463.2007.00722.x.

Boyd, D. A., Tyler, S., Christianson, S., et al. 2004. Complete nucleotide sequence of a 92-kilobase plasmid harboring the CTX-M-15 extended-spectrum beta-lactamase involved in an outbreak in long-term-care facilities in Toronto, Canada. *Antimicrob Agents Chemother*, 48(10), 3758-3764. <https://doi.org/10.1128/AAC.48.10.3758-3764.2004>.

Khalifa, H. O., Oreiby, A. F., Abd El-Hafeez, A. A., Abd El Latif, A., Okanda, T., Kato, Y., & Matsumoto, T. 2021a. High β-lactam and quinolone resistance of *Enterobacteriaceae* from the respiratory tract of sheep and goats with respiratory disease. *Animals*, 11(8), 2258.
